# Ultrafast single-qubit gates through multi-photon transition removal

**DOI:** 10.1038/s41467-026-78373-4

**Published:** 2026-10-23

**Authors:** Y. Gao, A. Galicia, J. D. Da Costa Jesus, Y. Liu, Y. Haddad, D. A. Volkov, J. R. Guimarães, H. Bhardwaj, M. Jerger, M. Neis, B. Li, F. A. Cárdenas-López, F. Motzoi, P. A. Bushev, R. Barends

**Affiliations:** 1 Institute for Functional Quantum System (PGI-13), Forschungszentrum Jülich, Jülich, Germany; 2 Department of Physics, RWTH Aachen University, Aachen, Germany; 3 Institute for Quantum Control (PGI-8), Forschungszentrum Jülich, Jülich, Germany; 4 Institute for Theoretical Physics, University of Cologne, Cologne, Germany

**Keywords:** Quantum information, Qubits

## Abstract

Fast qubit control is essential for scalable quantum computing. However, the strong driving that is required can induce significant leakage from the computational subspace, particularly in weakly anharmonic qubits. We demonstrate single qubit gates with a total leakage error consistently below 2.0 × 10^−5^ on a transmon qubit, and obtain fidelities above 99.98% at 6.8 ns pulse duration in both *X* and *X*/2 gates. This is achieved by removing direct transitions beyond nearest-neighbor levels using double recursive applications of Derivative Removal by Adiabatic Gate pulse shape. We also introduce the amplified leakage error sequence which can directly measure the leakage error down to 10^−6^. We test single qubit gates across a range of pulse durations using multiple benchmarking methods to reveal the error channels. The presented approach can be straightforwardly adapted by other platforms.

## Abstract (web summary)

Fast quantum gates are often limited by leakage from the computational states into higher energy levels. Here, the authors demonstrate high fidelity superconducting qubit gates with durations down to 6.8 ns by suppressing previously overlooked multi photon transitions, achieving leakage errors below 2 × 10^−5^.

## Introduction

One of the main enablers in quantum computing is having qubit control that is precise and fast^1–4^. However, qubits typically have multilevel structures^5–8^, making them prone to unwanted transitions from fast gates. This leakage out of the computational subspace is especially detrimental to algorithms, as it has been observed to cause long-lived errors, such as in quantum error correction^9–11^. This forces a choice between either achieving fast gates or having low leakage. Previous works focus on suppressing leakage by mitigating the first-to-second excited-state transition, overlooking multi-photon transitions, and achieving faster gates with further reductions in leakage has remained elusive.

Superconducting qubits are engineered quantum devices that have multiple energy levels, distinct from natural two-level systems. Therefore, overcoming undesired transitions has been a constant challenge in the quantum control of such devices^12–16^. The first work to mitigate this leakage used quadrature modulation to direct the quantum evolution, using the Derivative Removal by Adiabatic Gate (DRAG) method^13^, which has since become standard practice. Later works have explored methods ranging from frequency domain optimization to piecewise constant pulse engineering^17–20^. What has seemingly gone unrecognized is that when pushing for performance with ever faster gates, the qubit enters the strongly driven regime where the driving strength approaches the anharmonicity, altering gate physics.

Here, we demonstrate single-qubit gates with a total leakage error consistently below 2.0 × 10^−5^, and obtain fidelities above 99.98% for pulse durations down to 6.8 ns for both *X* and *X*/2 gates. This is achieved by removing direct transitions beyond nearest-neighbor levels using a double recursive implementation of the DRAG method, which we name the R2D method. Moreover, we find that at such short gate durations and strong driving strengths, the main error source is from these higher-order transitions. This is all shown in the widely-used superconducting transmon qubit^7,21^, which has a weakly anharmonic level structure and suffers significantly from higher-order transitions. We also introduce an approach for amplifying leakage error that can precisely quantify leakage rates below 10^−6^. The presented approach can be readily applied to other qubit types as well.

## Results

### Multi-photon transitions in the strongly driven regime

Higher-order transitions beyond nearest-neighbor energy levels are normally either considered forbidden or can be safely neglected for weak driving. Yet, further leakage reduction is only possible by addressing these transitions, which appear during the evolution and can be revealed through the Schrieffer-Wolff (S-W) transform ^22^. We start by describing the system in the rotating frame at the microwave-driven frequency *ω*_*d*_. The Hamiltonian of a capacitively driven qubit can be written as ^13^,

1 $$H/\hbar={\sum}_{j}{\delta }_{j}\left\vert j\right\rangle \left\langle j\right\vert+{\lambda }_{j+1}\left(\dfrac{{\Omega }_{R}}{2}{\sigma }_{j,j+1}^{x}+\dfrac{{\Omega }_{I}}{2}{\sigma }_{j,j+1}^{y}\right),$$

where *δ*_*j*_ = *ω*_*j*_ − *jω*_*d*_ is the detuning of the *j*th energy level. Ω  = Ω_*R*_ + *i*Ω_*I*_ is the control pulse, ${\sigma }_{j,k}^{x}=\left\vert j\right\rangle \left\langle k\right\vert+\left\vert k\right\rangle \left\langle j\right\vert$ and ${\sigma }_{j,k}^{y}=i\left\vert k\right\rangle \left\langle j\right\vert -i\left\vert j\right\rangle \left\langle k\right\vert$ are the Pauli operators, and *λ*_*j*_ is the relative strength of different transitions, with ${\lambda }_{j}\approx \sqrt{j}$. When the system is driven at resonance: *ω*_*d*_ = *ω*_*q*_ = *ω*_1_, and *δ*_2_ = *ω*_2_ − 2*ω*_1_ = *Δ* is the anharmonicity. In this qubit frame, the leakage from $\left\vert 1\right\rangle$ to $\left\vert 2\right\rangle$ can be straightforwardly identified through the ${\sigma }_{1,2}^{x}$ term. However, during the qubit driving, the qubit eigenstates are modified by the driving field, which leads to a superposition of higher qubit states, as well as shifts in eigenfrequencies. Therefore, to fully minimize the leakage out of the computational subspace with faster and stronger drives, we have to consider higher leakage levels and look at the dressed states of the coupled system formed by the qubit and the driving field.

**Fig. 1:**
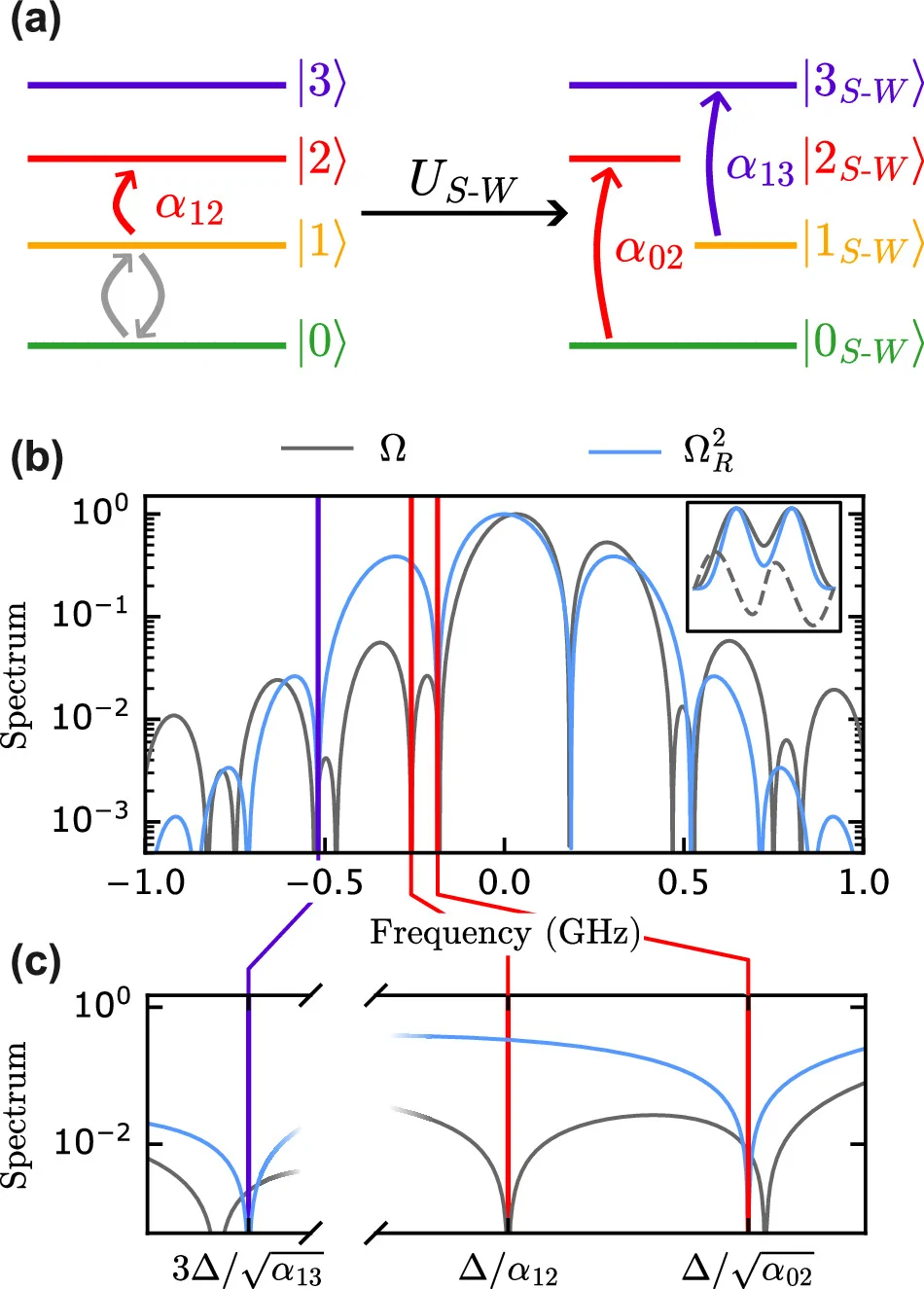
Schematic of qubit level structures and pulse shapes for fast gates. **a** Schematic representation of qubit energy levels in the qubit eigenbasis (left) and in the dressed eigenbasis under Schrieffer-Wolff transformation (right). The colored arrows show the unwanted leakage transitions. **b** An example R2D pulse for *t*_*p*_ = 7 ns. The magnitude spectrum of both Ω = Ω_*R*_ + *i*Ω_*I*_ (gray) and ${\Omega }_{R}^{2}$ (blue) are plotted. The vertical solid lines highlight the unwanted transition frequencies to $\left\vert 2\right\rangle$ (red) and $\left\vert 3\right\rangle$ (purple). The inset shows the normalized time-domain pulse for both Ω and ${\Omega }_{R}^{2}$, and the dashed line shows Ω_*I*_. **c** The same magnitude spectrum of Ω and ${\Omega }_{R}^{2}$ as in (**b**), highlighting the frequencies of the unwanted single- and multi-photon transitions.

We now use the S-W transform on the system Hamiltonian. Using ${U}_{S{\mbox{-}}W}=\exp (-i{\Omega }_{R}{\sum }_{j=0}^{1}{\lambda }_{j+1}{\sigma }_{j,j+1}^{y}/2\Delta )$, and keeping computationally relevant terms to the second order in Ω_*R*_/*Δ* we get ^23^

2 $$\begin{array}{l}{H}_{S{\mbox{-}}W}/\hbar\approx \dfrac{{\Omega }_{R}}{2}{\sigma }_{0,1}^{x}+\dfrac{{\Omega }_{I}+\dfrac{{\dot{\Omega }}_{R}}{\Delta }}{2}\left({\sigma }_{0,1}^{y}+{\lambda }_{2}{\sigma }_{1,2}^{y}\right) \\ \qquad\qquad\quad+ \left({\delta }_{1}+\dfrac{4-{\lambda }_{2}^{2}}{4\Delta }{\Omega }_{R}^{2}\right)\left\vert 1\right\rangle \left\langle 1\right\vert+\dfrac{{\lambda }_{2}}{8\Delta }{\Omega }_{R}^{2}{\sigma }_{0,2}^{x}+\dfrac{{\lambda }_{2}{\lambda }_{3}}{8\Delta }{\Omega }_{R}^{2}{\sigma }_{1,3}^{x},\end{array}$$

where the ${\sigma }_{0,2}^{x}$ and ${\sigma }_{1,3}^{x}$ terms in Eq. (2) reveal the direct transitions between higher levels. See Fig. 1(a) for a schematic representation. Interestingly, those transitions violate selection rules in the qubit frame. The two-photon nature of the transitions is further underlined by the squared term, ${\Omega }_{R}^{2}$. Moreover, the quadratic dependence of the amplitude emphasizes its importance when the pulse duration is short.

With higher-order transitions identified, we propose a pulse shape that suppresses the frequency spectrum of ${\Omega }_{R}^{2}$ at the relevant two-photon transition frequencies. Given a control envelope ansatz Ω_0_, we recursively apply the DRAG technique to remove the multi-photon transitions. To keep ${\Omega }_{R}^{2}$ real and avoid phase errors, we use the second derivative to remove the frequency components. A detailed proof and modeling are given in Ref. ^23^. We also include parameters *α*_02_ and *α*_13_ which allow for fine-tuning the spectral notches during in-situ optimization,

3 $${\Omega }_{R}^{2}=\left(1+\dfrac{{\alpha }_{02}}{{\Delta }^{2}}\dfrac{{d}^{2}}{d{t}^{2}}\right)\left(1+\dfrac{{\alpha }_{13}}{9{\Delta }^{2}}\dfrac{{d}^{2}}{d{t}^{2}}\right){\Omega }_{0}^{2}.$$

Subsequently, we apply the standard single-photon DRAG correction and include *α*_12_,

4 $$\Omega=\left(1-i\dfrac{{\alpha }_{12}}{\Delta }\dfrac{d}{dt}\right){\Omega }_{R}.$$

Similar to Ref. ^24^, we include a constant detuning to correct the phase error. To ensure that the derivatives exist and the pulse spectrum is finite, the envelope ansatz Ω_0_ must be smooth to at least 3rd order and Ω(0) = Ω(*t*_*p*_) = 0, where *t*_*p*_ is the pulse length. In this work, we use ${\Omega }_{0}=A{\sin }^{4}(\pi t/{t}_{p})$ and we add 1 ns padding after the pulse to account for possible pulse distortions in the system ^25^. Using the Fourier transform, the pulse correction results in,

5 $$\left[{\Omega }_{R}^{2}\right]\left(\omega \right)=\left(1-\dfrac{{\alpha }_{02}{\omega }^{2}}{{\Delta }^{2}}\right)\left(1-\dfrac{{\alpha }_{13}{\omega }^{2}}{9{\Delta }^{2}}\right)\left[{\Omega }_{0}^{2}\right]\left(\omega \right),$$

6 $$\Omega \left(\omega \right)=\left(1-\dfrac{{\alpha }_{12}\omega }{\Delta }\right){\Omega }_{R}\left(\omega \right).$$

The effect of the R2D pulse shaping can be intuitively understood in the frequency domain of both Ω and ${\Omega }_{R}^{2}$ [Fig. 1b, c], where we null the signal spectrum at leakage transition frequencies.

We experimentally validate the R2D pulse shape using an isolated transmon qubit (see Supplementary Note 1). The qubit is operated at the flux-insensitive spot where it has a frequency *f*_*q*_ of 5.323 GHz and an anharmonicity *Δ*/2*π* of -225 MHz.

### Amplified leakage error

We start the validation by introducing a method capable of measuring a leakage error down to 10^−6^. The standard approach uses leakage randomized benchmarking (LRB) ^26^, where the leakage is measured as it accumulates during Clifford-based randomized benchmarking. However, this approach has drawbacks. First, the random nature causes the accumulation of higher-level state population to be incoherent. Second, extracting the leakage error of a specific gate requires interleaving, which can become expensive in terms of time and measurements. Third, since LRB accumulates leakage incoherently, it becomes challenging to distinguish coherent gate leakages from thermal transitions. Moreover, we show later that it becomes difficult to obtain precision below 10^−5^ using LRB. Therefore, we introduce an amplified leakage error (ALE) method that coherently accumulates leakage errors for both *X* and *X*/2 gates, allowing for highly accurate measurements.

**Fig. 2:**
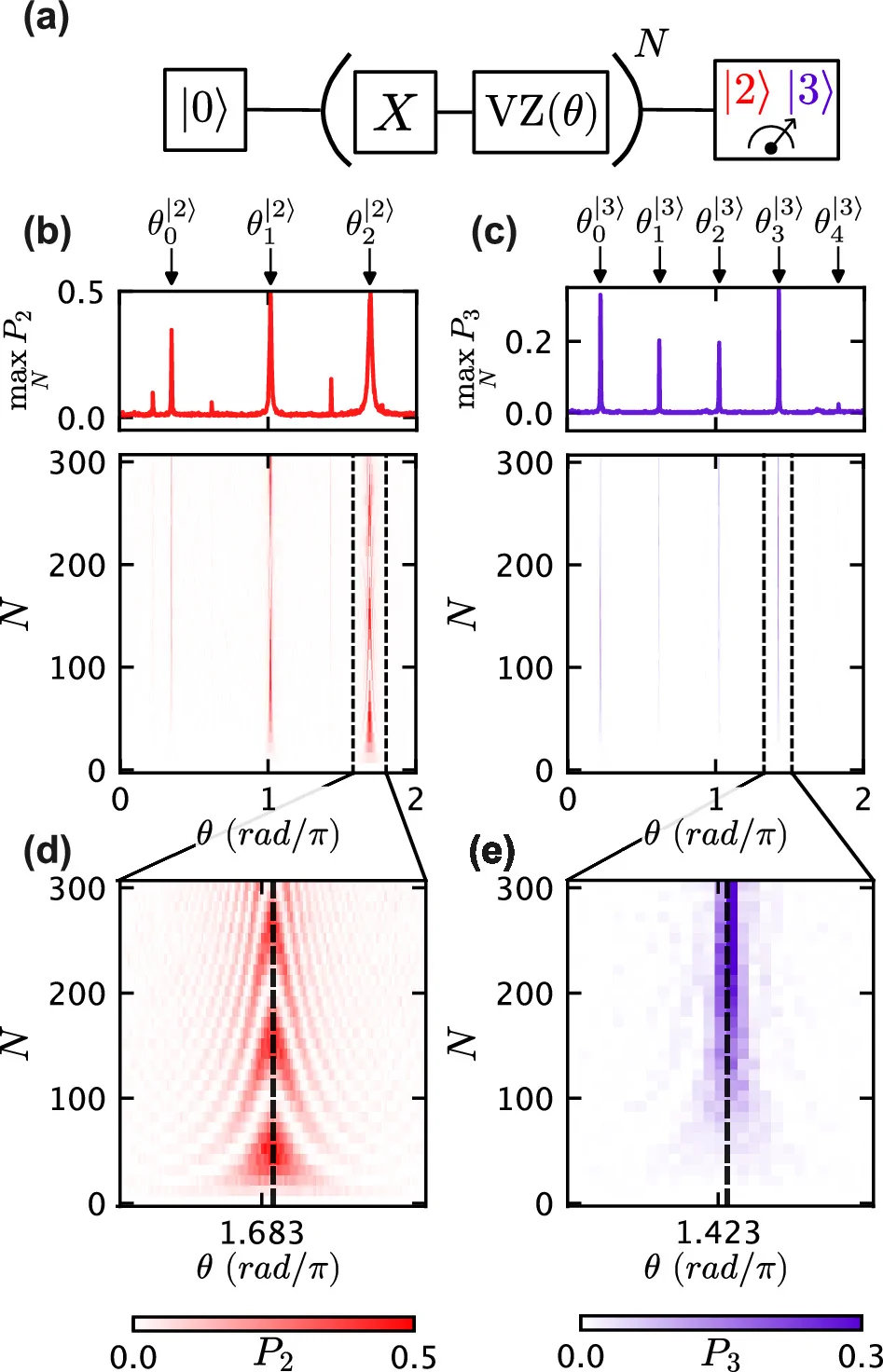
Amplified leakage error experiments. **a** The schematic of the ALE pulse sequence. We repeatedly apply *N* times an *X* operation followed by a VZ with angle *θ*. The sequence ends with measurements of the leakage population. **b** The $\left\vert 2\right\rangle$ leakage population as a function of *θ* and *N*. The line plot on top shows the maximum leakage population for each *θ*. There are three amplification phases, which are located at ${\theta }_{k}^{\left\vert 2\right\rangle }=(0.35+2k/3)\pi,\,k=0,1,2$, and evenly spaced in 2*π* period. Other peaks are caused by measurement error. **c** Same as (**b**) but plotted with $\left\vert 3\right\rangle$ leakage population. There are five amplification phases, which are located at ${\theta }_{k}^{\left\vert 3\right\rangle }=(0.22+2k/5)\pi,\,k=0,1,2,3,4$, and evenly spaced in 2*π* period. The peak at ${\theta }_{4}^{\left\vert 3\right\rangle }$ is low, as it has not reached its oscillation period within 300 gates. **d**,**e** The same data as in (**b**,**c**) but with a magnified window around one of the amplification phases (dashed lines). The clear chevron pattern indicates a coherent oscillation of the leakage population.

The key notion behind the ALE sequence is the phase matching of the leakage population after each operation. Using a *Z*-rotation with the correct angle, the population will be phase-matched and coherently accumulated. The ALE sequence consists of *N* repetitions of an *X* or composite *X* operation and a virtual-*Z* (VZ) gate with angle *θ* [Fig. 2a]. A similar principle was previously employed to amplify errors in two-qubit gates ^27,28^ and for *X*/2 gate calibration ^19^. The unitary of *X* containing coherent leakages and the VZ can be written as

7 $$X=\left(\begin{array}{ccc}0&1&{A}_{1}{e}^{-i({\phi }_{1}+{\phi }_{2})}\\ 1&0&{A}_{0}{e}^{-i({\phi }_{0}+{\phi }_{2})}\\ -{A}_{0}{e}^{i({\phi }_{0}-{\phi }_{2})}&-{A}_{1}{e}^{i({\phi }_{1}-{\phi }_{2})}&{e}^{-i2{\phi }_{2}}\end{array}\right),$$

8 $${{\rm{VZ}}}(\theta )=\left(\begin{array}{ccc}1&0&0\\ 0&{e}^{-i\theta }&0\\ 0&0&{e}^{-i2\theta }\end{array}\right),$$

where *A*_0_, *A*_1_ ≪ 1 are magnitudes of the leakage population transferred to $\left\vert 2\right\rangle$, *ϕ*_0_ and *ϕ*_1_ are phases of the leakage population, and 2*ϕ*_2_ is the phase acquired in $\left\vert 2\right\rangle$ during *X*. The leakage rate of *X* is defined as ${\epsilon }_{leak,\left\vert 2\right\rangle }=({A}_{0}^{2}+{A}_{1}^{2})/2$. After we examine the ALE sequence, we find (see Supplementary Note 6) that leakage is coherently accumulated only when *θ* satisfies

9 $$3\theta+4{\phi }_{2}=2k\pi,\,k\in {\mathbb{Z}}.$$

At the phase ${\theta }_{k}^{\left\vert 2\right\rangle }=(2k\pi -4{\phi }_{2})/3$, the qubit population coherently oscillates between the computational subspace and $\left\vert 2\right\rangle$, and ${P}_{2}={a}_{k}{\sin }^{2}({\omega }_{k}N/2)$, where *a*_*k*_ and *ω*_*k*_ are the amplitude and the frequency of the oscillation at amplification phases ${\theta }_{k}^{\left\vert 2\right\rangle }$. The leakage error of *X* is proportional to the weighted sum of squared oscillation frequencies at all amplification phases,

10 $${\epsilon }_{leak,\left\vert 2\right\rangle }=\dfrac{1}{6}\mathop{\sum }_{k=1}^{3}{a}_{k}{\omega }_{k}^{2}.$$

Similar analysis can be applied for the leakage rate to $\left\vert 3\right\rangle$, where five distinct phases exist ${\theta }_{k}^{\left\vert 3\right\rangle },\,k=0,1,2,3,4$ (see Supplementary Note 6). While the coherent leakage error caused by the control pulse is amplified, the thermal leakage with random phases will remain low. Therefore, the ALE sequence can selectively amplify the coherent leakage error to different states.

We show an example of the experimental implementation of the ALE sequence in Fig. 2b–e. The leakage population is plotted as a function of *N* and *θ*. The amplified leakage error to $\left\vert 2\right\rangle$ is on the left, and error to $\left\vert 3\right\rangle$ is on the right. Despite the existence of state preparation and measurement (SPAM) errors, the amplification of leakages to different states is independent and clearly visible. The line plots in Fig. 2b, c show the maximum leakage population over *N* as a function of *θ*. The three (five) amplification phases ${\theta }_{k}^{\left\vert 2\right\rangle }$ (${\theta }_{k}^{\left\vert 3\right\rangle }$) are evenly spaced in [0, 2*π*), and marked by arrows. The small peaks in Fig. 2b are caused by SPAM errors. Finally, in Fig. 2d, e, we see clear chevron patterns evidencing a coherent process, with dashed lines at target phases.

The ALE experiment used in the gate calibration procedure can be further simplified. In fact, the target phases satisfying Eq. (9) stay unchanged unless *ϕ*_2_ varies. As *ϕ*_2_ is dominated by the frame frequency of $\left\vert 2\right\rangle$, the target phases are stable in a large range of parameters for a constant pulse duration. Thus, only a 2*π*/3 window is needed to find all phases, and a narrow window around them can be used afterwards. This significantly reduces the calibration time needed in the closed-loop optimization.

The amplified leakage error sequence complements the metrology and calibration toolset for characterizing all errors of single-qubit gates, together with the amplified phase error (APE) sequence ^24,29–31^ and the amplified amplitude error (AAE) sequence ^32^. For each set of parameters (*α*_12_, *α*_02_, *α*_13_), APE and AAE experiments can deterministically find the optimal constant detuning and pulse amplitude (see Supplementary Note 8). Using the leakage error extracted from ALE experiments as the cost function, the Nelder-Mead optimization can reliably find the optimal gate under the R2D pulse shaping.

### Comparison between different benchmarking methods

**Fig. 3:**
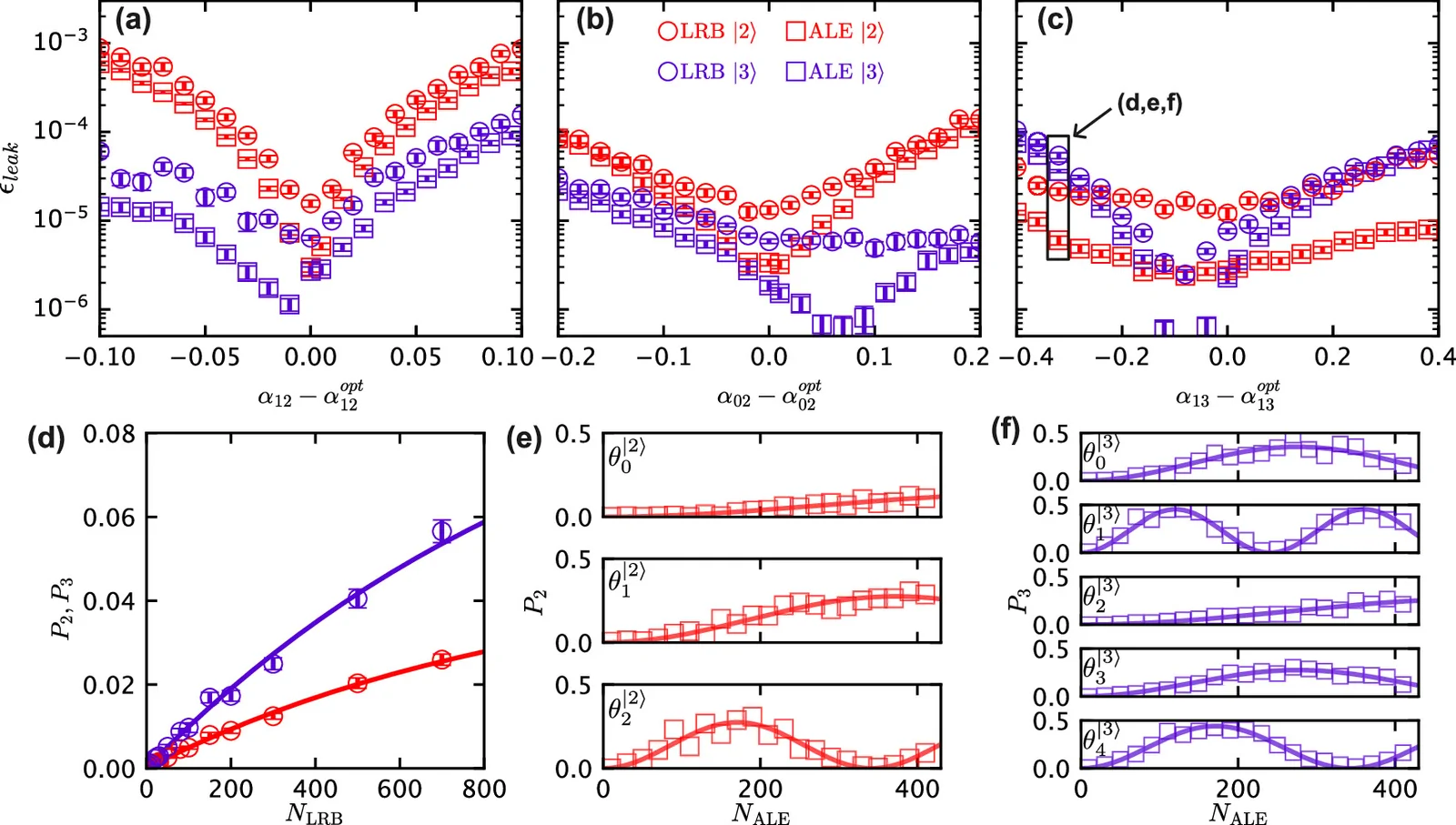
Extracting leakage error from leakage randomized benchmarking and amplified leakage error. a–c Leakage error rates of an X gate at *t*_*p*_ = 7 ns extracted from LRB (circle) and ALE (square) for leakage state $\left\vert 2\right\rangle$ (red) and $\left\vert 3\right\rangle$ (purple) as a function of parameter *α*_12_ (**a**), *α*_02_ (**b**), and *α*_13_ (**c**). The optimal parameter set $({\alpha }_{12}^{opt},{\alpha }_{02}^{opt},{\alpha }_{13}^{opt})$ is obtained through an in-situ optimization. Error bars are the standard error from the fit. The raw traces of the marked data points in (**c**) are shown. **d** Raw traces used to extract leakage errors marked in (**c**) for both states using LRB. Only the interleaved curves are shown. Solid lines are fit curves. Error bars are the standard error of the mean. **e**,**f** Raw traces used to extract leakage errors marked in (**c**) for $\left\vert 2\right\rangle$ (**e**) and $\left\vert 3\right\rangle$ (**f**) using ALE. The traces are obtained at the amplification phases shown in Fig. 2b, c. Solid lines are fit curves.

We validate the leakage errors obtained from the ALE through a comparison with the standard LRB method. In Fig. 3a–c, leakage errors of an *X* gate at *t*_*p*_ = 7 ns from both methods are plotted as a function of *α*_12_, *α*_02_, and *α*_13_, respectively. The parameters are individually swept around the optimal value obtained from an in-situ gate calibration, $({\alpha }_{12}^{opt},{\alpha }_{02}^{opt},{\alpha }_{13}^{opt})=(0.864,1.467,1.796)$. For each parameter set, APE and AAE experiments are used to find the minimum amplitude and phase error. A set of raw traces for the data points marked in Fig. 3c from both methods is shown in Fig. 3d–f. The incoherently accumulated leakage of interleaved LRB, averaged over 60 random sequences, as well as the solid fit lines, are plotted in Fig. 3d. The population stays below 0.06 even after 800 Clifford gates. On the other hand, the amplified leakages from ALE experiments for $\left\vert 2\right\rangle$ and $\left\vert 3\right\rangle$ show clear coherent oscillations at amplification phases within just 400 operations; see the fitted curves in Fig. 3e, f. As shown in Fig. 3a–c, we find that the leakage rates increase by almost two orders of magnitude when the parameters are biased away from the optimal value. While a good correspondence of the leakage rates between the two methods is prominently visible, noticeable differences appear for leakages below 10^−5^. Despite differences in leakage errors between methods, the minimum total leakage is achieved with the same pulse parameters.

The leakage behavior in Fig. 3 provides intuitive guidance for optimizing pulse parameters. After minimizing the phase and amplitude error, the leakage is solely controlled by (*α*_12_, *α*_02_, *α*_13_), and the optimal value can be obtained through the Nelder-Mead optimization. The corrections from *α*_02_ and *α*_13_ are nearly orthogonal, which benefits the optimization. Additionally, the ALE sequence requires fewer operations and shows larger leakage populations, which emphasizes its advantages in terms of time and measurements. Furthermore, a reliable gate optimization can be achieved through ALE instead of LRB, evidenced by the good correspondence in both leakage errors and the optimal parameter values. We attribute the differences in leakage errors to the fact that the ALE method is insensitive to thermal excitations and $\left\vert 2\right\rangle \to \left\vert 3\right\rangle$ transition errors (see Supplementary Note 6).

### Error budgets for single-qubit gates

In order to evaluate the gate performance from all aspects, we need additional benchmarking tools. Besides LRB characterizing the leakage error *ϵ*_*leak*_, we use standard randomized benchmarking (RB)^33^ to characterize the total gate error *ϵ*_*tot*_, and purity randomized benchmarking (PRB)^34^ to characterize the decoherence error *ϵ*_*decoh*_ (see Supplementary Note 9). All methods are run in an interleaved fashion to extract errors for the target gate.

**Fig. 4:**
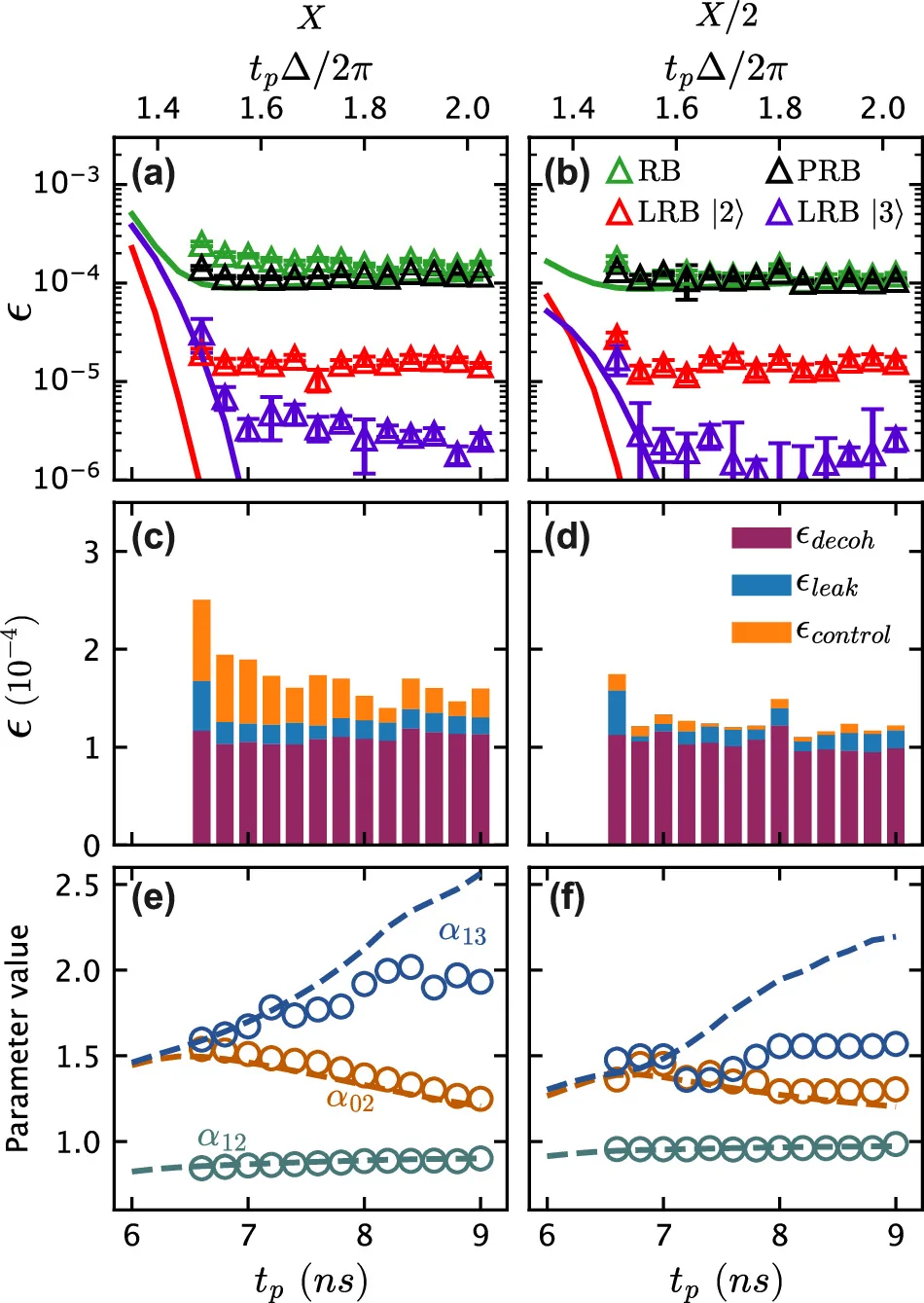
Gate benchmarks with varying pulse lengths. Gate performance with pulse durations *t*_*p*_ ∈ [6.6, 9] ns. X gate is on the left, and X/2 gate is on the right. **a**,**b** Error rates extracted from RB (green), PRB (black), LRB for $\left\vert 2\right\rangle$ (red), and LRB for $\left\vert 3\right\rangle$ (purple). Solid lines are simulation results. Error bars are the standard error from the fit. **c**,**d** Different contributions to total gate error, including decoherence error (dark magenta), leakage error (blue), and coherent control error (orange). **e**,**f** Parameter sets of optimal gates from in-situ calibrations (symbols) and simulations (dashed lines).

To fully demonstrate the performance of the R2D pulse shaping, we perform a complete benchmark of single-qubit gates with varying pulse lengths, and construct the error budgets. Using the calibration procedure discussed previously, we bring up both *X* and *X*/2 gates with pulse length *t*_*p*_ ∈ [6.6, 9] ns. Pulses shorter than 6.6 ns will result in an unstable gate optimization as negative values appear in ${\Omega }_{R}^{2}$, and pulses longer than 9 ns will cause benchmarking sequences to exceed the waveform memory limit of the control electronics. To ensure the best results, a constant phase correction is included in the pulse (see Supplementary Note 2) to compensate for the accumulated phase shift caused by the constant detuning. In Fig. 4, we demonstrate the benchmarking results of *X* gates on the left, and *X*/2 gates on the right. Error rates from all benchmarks are shown in Fig. 4a, b. We see that the total leakage error stays below 2 × 10^−5^ down to a pulse length of 6.8 ns. At a pulse duration of 6.6 ns, the leakage error to $\left\vert 3\right\rangle$ starts to dominate. At a pulse duration of 6.8 ns, we achieve *X* and *X*/2 gates with a total gate error of (1.9 ± 0.1) × 10^−4^ and (1.1 ± 0.1) × 10^−4^, respectively. We note that, with *t*_*π*_ being the *π*-pulse duration, this is equivalent to a normalized driven strength of Ω/*Δ* = *π*/*t*_*π*_*Δ* = 0.33, a record value for single-qubit gates in superconducting qubits. The solid green line shows the total gate error from a Lindblad master equation simulation. The solid purple line shows the simulated coherent leakage error to $\left\vert 3\right\rangle$ in the absence of incoherent processes. The same simulated leakage error to $\left\vert 2\right\rangle$ is plotted in red. We use the experimental results to separate the total gate error into constituents: *ϵ*_*tot*_ = *ϵ*_*decoh*_ + *ϵ*_*leak*_ + *ϵ*_*control*_, see Fig. 4c, d. We find that gate errors of both *X* and *X*/2 are mainly limited by decoherence errors around a level of 1.04 × 10^−4^, and coherent control errors increase with decreasing pulse lengths for *X* gates. We also show the optimal parameters for different pulse lengths in Fig. 4e, f, where symbols and dashed lines represent values from the in-situ calibration and the simulation, respectively.

In addition, when comparing the R2D pulse method, we find it outperforms the standard single-photon DRAG correction (see Supplementary Fig. 14), achieving a leakage reduction exceeding a factor of 20.

The data in Fig. 4 show that the R2D pulse shape enables gates with nearly 99.99% fidelity at the normalized driven strength Ω/*Δ* up to 0.33, limited by qubit decoherence. First, the leakage errors remain below 2.0 × 10^−5^ over a large domain, and the increase in leakage errors below a pulse length of 6.8 ns is well predicted by the simulated coherent leakage. This evidently demonstrates the importance of accounting for multi-photon leakages in single-qubit gates. We attribute the lower bound of leakages to excitations dominated by the voltage noise of room-temperature electronics for the microwave control. Second, both *X* and *X*/2 gates show small coherent errors, with the error being substantially below 1.0 × 10^−4^ for *X*/2 gates. For *X* gates, we attribute the coherent error increase with decreasing duration to microwave distortions, as it becomes more noticeable for shorter gates and higher amplitudes. This can be mitigated by introducing a small padding after the pulse. Third, the optimal pulse parameters from simulation and in-situ calibration compare well. Parameter *α*_13_ differs more for longer pulses because the change in $\left|3\right\rangle$ leakage is below the measurement threshold. We note that the correction of *α*_13_ can be omitted without sacrificing the performance at longer pulse durations for a certain leakage threshold; see Ref. ^23^ for simulation results in such cases. The simulated and experimental results being consistent in all aspects validates the R2D pulse shape.

## Discussion

In conclusion, we demonstrate fast and precise *X* and *X*/2 gates through suppression of multi-photon transitions in the strongly driven regime using only three parameters. This is the first control solution for the removal of multi-photon transitions in any quantum experimental setting, and it can be straightforwardly adapted by other qubit platforms or subfields of quantum physics where such transitions are ubiquitous. We also introduce the ALE sequence, which allows precision near 10^−6^ in coherent leakage characterization, and complements the metrology toolset for the single-qubit gate calibration. In large-scale processors, the resulting ultrafast single-qubit gates could further reduce the decoherence error accumulated by idling qubits, improving the overall algorithmic performance. In addition, the ability to suppress multiple transitions is also beneficial for mitigating the microwave crosstalk. Moreover, the same approaches can be used to mitigate the leakage in fast two-qubit gates. These results set a new benchmark for single-qubit gates in terms of both fidelities and speed, and with enhanced qubit coherence and precise distortion characterization, the proposed methodology opens a path to achieving gates with a fidelity of 99.999%.

## Methods

For a detailed description of the methods used, see the Supplementary Information.

## Supplementary information

Supplementary Information

Transparent Peer Review file

## Data Availability

The data that support the findings of this study are available from the corresponding author upon request.
