# Supplementary material for: Ultrafast single-qubit gates through multi-photon transition removal
Source: Nat Commun. 2026 Oct 23;17:10874. doi: 10.1038/s41467-026-78373-4 (PMC13694273; doi:10.1038/s41467-026-78373-4)
Supplement: Supplementary file 1 [file 41467_2026_78373_MOESM1_ESM.pdf]

Supplementary Materials for "Ultrafast Single Qubit Gates through Multi-Photon Transition Removal"

Y. Gao (高源),[1,2] A. Galicia,[1,2] J. Da Costa Jesus,[3,4] Y. Liu (刘业彬),[1] Y. Haddad,[1,2] D. A. Volkov,[1,2] J. R. Guimarães,[1,2] H. Bhardwaj,[1,2] M. Jerger,[1] M. Neis,[1,2] B. Li (李伯熙),[3] F. A. Cárdenas-López,[3] F. Motzoi,[3,4] P. A. Bushev,[1] and R. Barends[1,2]

[1]*Institute for Functional Quantum System (PGI-13), Forschungszentrum Jülich, 52425 Jülich, Germany*
[2]*Department of Physics, RWTH Aachen University, 52074 Aachen, Germany*
[3]*Institute for Quantum Control (PGI-8), Forschungszentrum Jülich, 52425 Jülich, Germany*
[4]*Institute for Theoretical Physics, University of Cologne, 50937 Cologne, Germany*

(Dated: September 4, 2026)

SUPPLEMENTARY NOTE 1: QUBIT PARAMETERS AND STATE DISCRIMINATION

In this work, we use an isolated tunable transmon qubit that does not couple to any other qubits or couplers. The qubit chip is fabricated using the E-beam lithography. The Al-Al_2O_3-Al junctions are defined using the standard Manhattan process on a Si substrate.

The qubit has $E_C/h = 204.8$ MHz and $E_J/h = 18.7$ GHz. The operating point is near the flux insensitive point, where the qubit is at the frequency $f_q = 5.323$ GHz and has anharmonicity $\Delta/2\pi = -225.0$ MHz. The qubit XY control line is capacitively coupled to the qubit, and the coupling capacitance is $C_c = 95$ aF. The qubit is dispersively readout from a quarter-wave resonator. The resonator is at the frequency $f_r = 6.799$ GHz, and has a qubit-resonator coupling of $g_{qr}/2\pi = 45$ MHz. The average qubit coherence properties are $T_1 = 40$ μs from T_1 experiments, $T_2^E = 56$ μs from Echo experiments, and $T_2^R = 45$ μs from Ramsey experiments.

To better quantify the leakage rate to different states, we use four-state discrimination. We reset the qubit to $|0\rangle$ by relaxation, and measure $2^{17} = 131072$ shots in each state. The acquisition length is 2.048 μs. The raw IQ points and the fitted 2D Gaussian distributions are shown in Supplementary Fig. 1. To discriminate the readout data, the nearest center method is used. We obtain the assignment matrix as

$$\Lambda = \begin{pmatrix} 89.64 & 9.49 & 0.75 & 0.12 \\ 13.10 & 86.03 & 0.80 & 0.07 \\ 13.67 & 11.43 & 69.93 & 4.97 \\ 10.46 & 2.76 & 15.78 & 71.00 \end{pmatrix} \times 10^{-2}, \quad (1)$$

where Λ_{ij}, the element at i-th row and j-th column, indicates the probability of measuring $|j\rangle$ when qubit is prepared at $|i\rangle$. In this assignment matrix, a large number of thermal population $\bar{n} \approx 0.1$ is observed. We use postselection to reduce the initialization error, and the new assignment matrix is

$$\Lambda^{ps} = \begin{pmatrix} 97.793 & 2.129 & 0.073 & 0.007 \\ 7.873 & 91.604 & 0.519 & 0.004 \\ 6.726 & 11.173 & 77.602 & 4.499 \\ 3.116 & 2.082 & 15.958 & 78.843 \end{pmatrix} \times 10^{-2}. \quad (2)$$

In all experiments performed in this work, we use postselection to reset the qubit to $|0\rangle$, and we do not correct for SPAM errors.

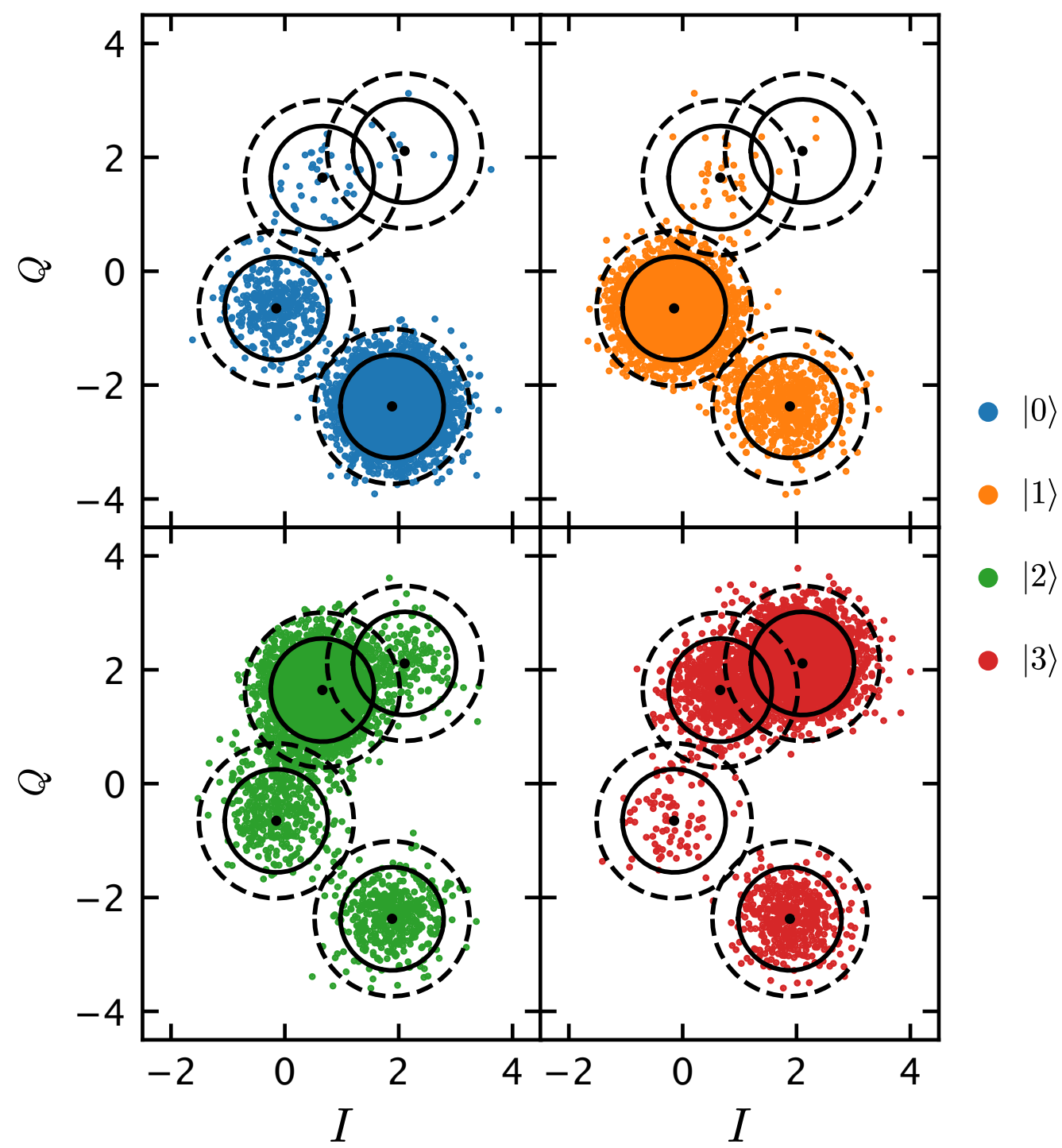

Supplementary Fig. 1. **Qubit readout.** Raw IQ points from the single shot experiment where qubit is prepared in the $|0\rangle$ (blue), $|1\rangle$ (orange), $|2\rangle$ (green), and $|3\rangle$ (red) states. We show 5000 out of 2^{17} preparations for each state. The black points are the fitted state centers, the circles have the radii corresponding to two (solid line) and three (dashed line) standard deviations of the data.

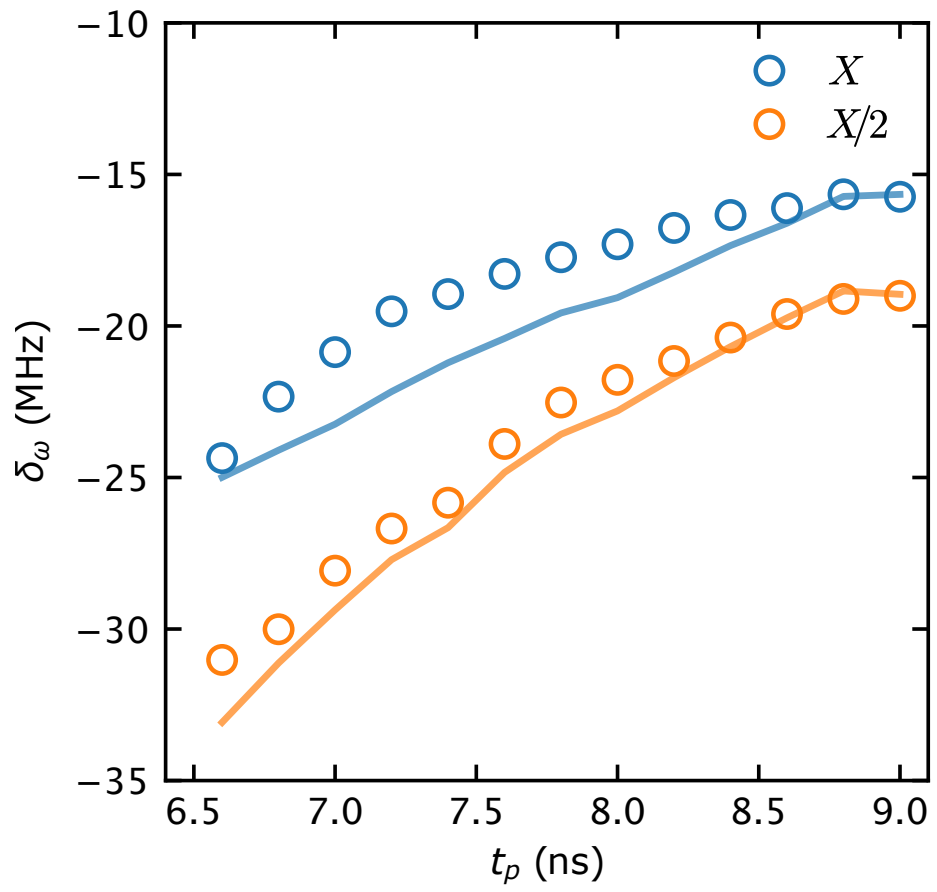

Supplementary Fig. 2. **Theoretical and experimental constant detune values.** Constant detuning corrections for different pulse durations from experiments (symbols) and predictions using the experimentally calibrated α_{12} (lines).

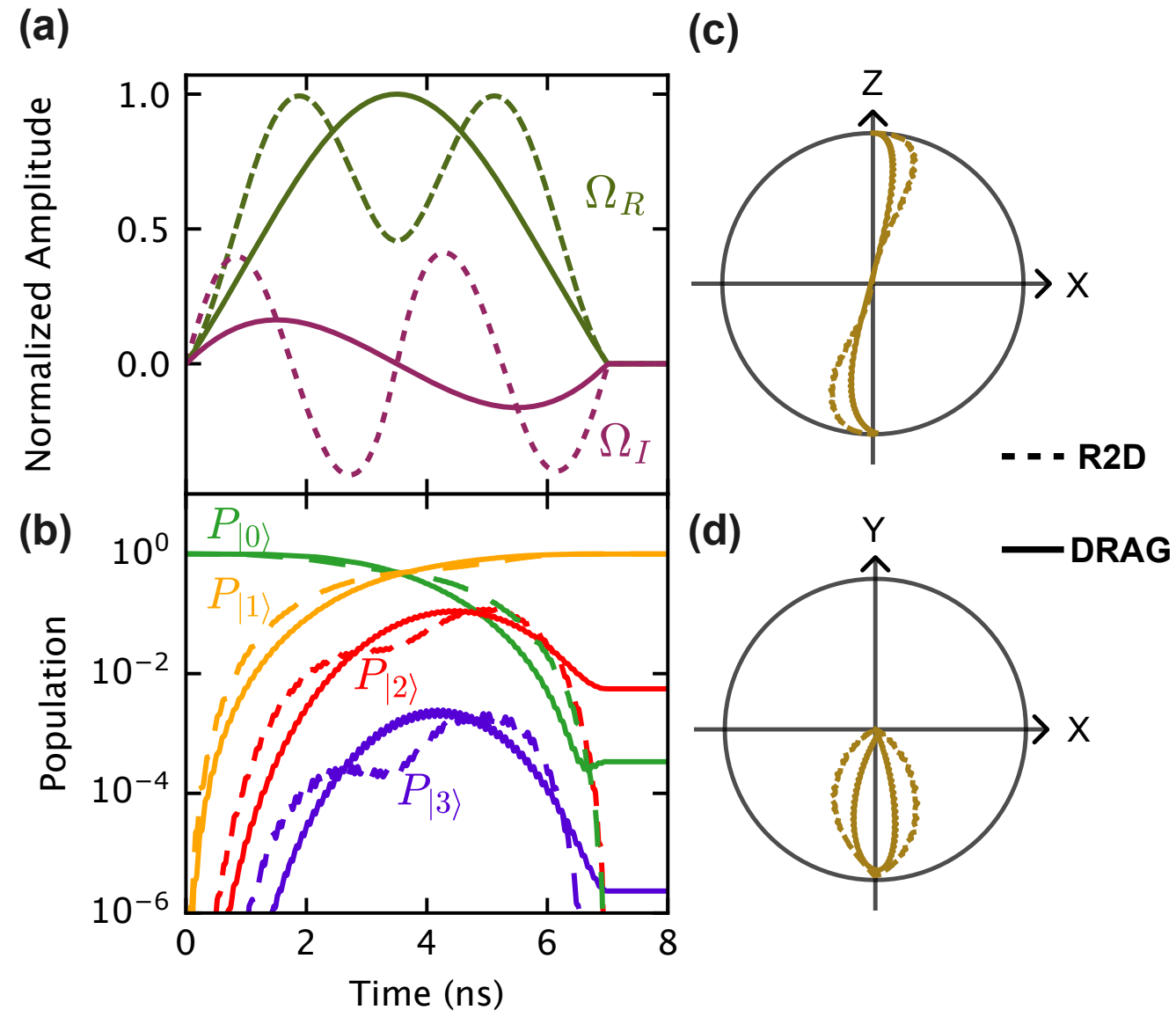

Supplementary Fig. 3. **Qubit Hamiltonian simulation.** Simulation results of the optimal pulses using R2D (dashed lines) and DRAG (solid lines) pulse shapes. (a) The time domain signals of the real part and the imaginary part. (b) Simulated population of lowest four states. (c) Qubit state trajectories projected in X-Z plane. (d) Qubit state trajectories projected in X-Y plane.

SUPPLEMENTARY NOTE 2: CONSTANT DETUNING AND PHASE CORRECTION

In this work, we use a constant detuning to correct the phase error. The theoretical value for the constant detuning δ_ω satisfies

$$\delta_\omega = 0.712 \frac{(4\alpha_{12} - 2)}{\Delta} \frac{\pi^2}{t_g^2}, \tag{3}$$

see Ref. [1] for detailed derivations. It matches well with experimental results [Supplementary Fig. 2], and can be used as initial values for the pulse. Additional to the constant detuning correction, an extra phase correction is included to compensate the accumulated phase shift,

$$\Omega'(t) = \Omega(t) e^{-i\delta_\omega (t - t_p/2)}, \tag{4}$$

where $\Omega(t)$ is the pulse envelope as in Eq. (4) in the main text. The phase correction $\delta_\omega t_p/2$ is particularly important when other single qubit gates of the same qubit are at different lengths, or their phase errors are corrected by other approaches. In the absence of this phase correction, the effective rotation axis will be away from x axis in the $x - y$ plane. This explains the issue reported in Ref. [2] that the best results are only achievable by using the same detuning and the same length for X and $X/2$ gates.

SUPPLEMENTARY NOTE 3: SINGLE QUBIT GATE SIMULATION

We simulate the qubit evolution without RWA, as the rotating wave approximation (RWA) is only valid under weak coupling [3]. The bare transmon is modeled in the qubit charge basis as

$$H_q = 4E_C \hat{n}^2 + E_J \cos\hat{\phi}, \tag{5}$$

where $\hat{n}$ is the charge operator, $\hat{\phi}$ is the phase operator, and

$$\cos\hat{\phi} = \frac{1}{2}\sum_n |n\rangle\langle n+1| + |n+1\rangle\langle n|. \tag{6}$$

Transmon parameters E_C and E_J are set such that the lowest four energy levels compare well with experiment results. There are in total 19 charge states in the simulation. A capacitively coupled microwave drive is modeled as

$$H_d = \hbar\Omega_{RF}(t)\hat{n}. \tag{7}$$

Microwave distortion is not considered. We also include 1 ns padding after the pulse as in the experiment. The pulse is modulated by the qubit frequency obtained from Supplementary Eq. (5). The QuTiP [4] package is used to simulate the qubit evolution. In Supplementary Fig. 3, simulation results of the optimal pulses at $t_p = 7$ ns using R2D and DRAG pulse shapes are shown.

To incorporate incoherent processes during the evolution, we use Lindblad master equation,

$$\dot{\rho} = \frac{i}{\hbar}[\rho, H_q + H_d] + \sum_i L_i \rho L_i^\dagger - \frac{1}{2}\left\{\rho, L_i^\dagger L_i\right\} \tag{8}$$

where ρ is the density operator, and we use collapse operators $L_- = \sqrt{\Gamma_\downarrow}a$, $L_+ = \sqrt{\Gamma_\uparrow}a^\dagger$, and $L_\phi = \sqrt{\Gamma_\phi}a^\dagger a$. $\Gamma_\downarrow = (1-\bar{n})/T_1$, $\Gamma_\uparrow = \bar{n}/T_1$, and $\Gamma_\phi = 1/T_2^E - 1/2T_1$ can be measured in experiments.

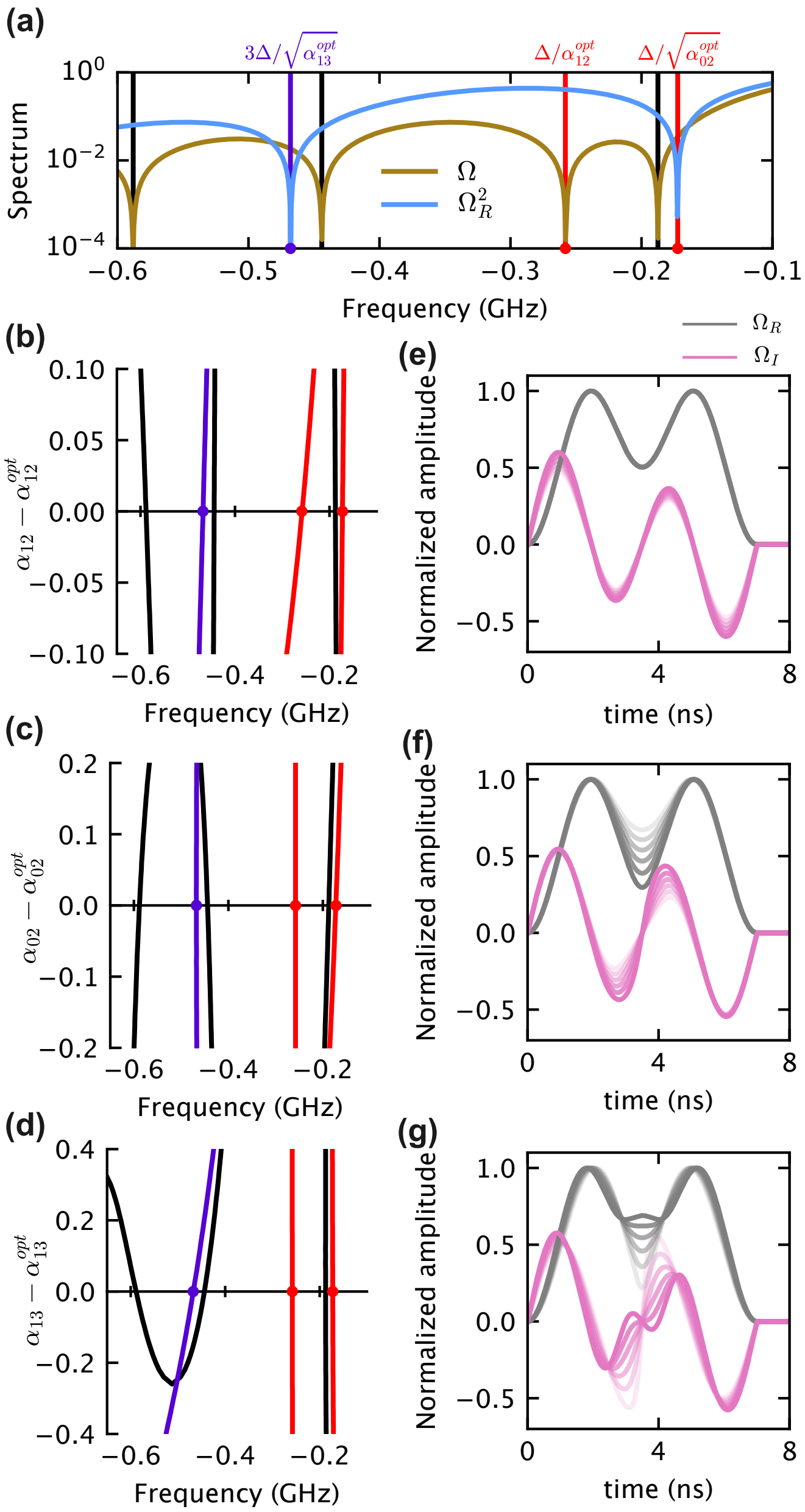

Supplementary Fig. 4. **R2D pulse shape in the frequency domain and the time domain.** (a) The optimal pulse at $t_p = 7$ ns with pulse parameter $(\alpha_{12}^{opt}, \alpha_{02}^{opt}, \alpha_{13}^{opt}) = (0.864, 1.467, 1.796)$. (b) The frequency domain pulses when parameter α_{12} is swept around α_{12}^{opt}. The colored lines are the notches shown in (a), and the corresponding points are marked with dots. (e) The time domain pulses when parameter α_{12} is swept around α_{12}^{opt}. The lighter color indicates a smaller value of the parameter. (c)(f) Same plots as in (b)(e) but sweeping α_{02}. (d)(g) Same plots as in (b)(e) but sweeping α_{13}.

SUPPLEMENTARY NOTE 4: PULSE SHAPE IN TIME AND FREQUENCY DOMAIN

It is important to understand in what way the corrections are affecting the pulse shape. In Supplementary Fig. 4, we show how the time and frequency domain pulses change when the pulse parameters are swept. In Supplementary Fig. 4(a), the spectrum of an optimal pulse at $t_p = 7$ ns is plotted, and the parameters $(\alpha_{12}^{opt}, \alpha_{02}^{opt}, \alpha_{13}^{opt}) = (0.864, 1.467, 1.796)$ are obtained from in-situ optimization. We mark the notches that we actively optimize with colors and the rest of notches black. The pulse parameters are swept individually around the optimal value and the pulses are plotted in both time and frequency domain. For time domain pulse, we use a lighter color to indicate a smaller value of the parameter. We sweep α_{12} in Supplementary Fig. 4(b)(e), α_{02} in Supplementary Fig. 4(c)(f), and α_{13} in Supplementary Fig. 4(d)(g). From the frequency domain notches, the corrections from α_{02} and α_{13} are nearly orthogonal, which agrees with the leakage rates scan in Fig. 3 in the main text. Note the opposite effects on $\Omega_R(t)$ as α_{02} and α_{13} increase, this enables a short pulse since larger α_{02} corrections in fast pulses tends to make Ω_R^2 negative.

SUPPLEMENTARY NOTE 5: EXPERIMENTAL SETUP AND SIGNAL DISTORTION

The experimental setup and an optical picture of the transmon are shown in Supplementary Fig. 5. The transmon qubit chip is packaged, and protected by a μ-metal shield. The device is mounted at the mixing chamber of a Bluefors XLD fridge. The qubit flux is directly biased by a Zürich Instruments HDAWG using the voltage offset of the channel. A Zürich Instruments SHFQA is used for the qubit readout. The readout channel has $f_{LO} = 7.0$ GHz, output range is 10 dBm, and input range is -10 dBm. The qubit XY control signal is generated by a Zürich Instruments SHFSG. We use $f_{LO} = 5.4$ GHz and output range of 10 dBm.

To mitigate the microwave signal distortion caused by the electronics and control wiring, we first measure the system transfer function from the electronics to the mixing chamber (MXC) of the fridge while the setup is in ambient condition. Then we apply the inverse of the transfer function to the sampled waveform, and upload the whole waveform of the experiment to the waveform memory in the electronics. This approach is significantly limited by the waveform memory of the electronics, which prohibits us to benchmark gates at longer pulse duration.

In Supplementary Fig. 6, we plot the measured transfer function of the system in the frequency domain. The bandwidth of ±1 GHz is necessary to address the distor-

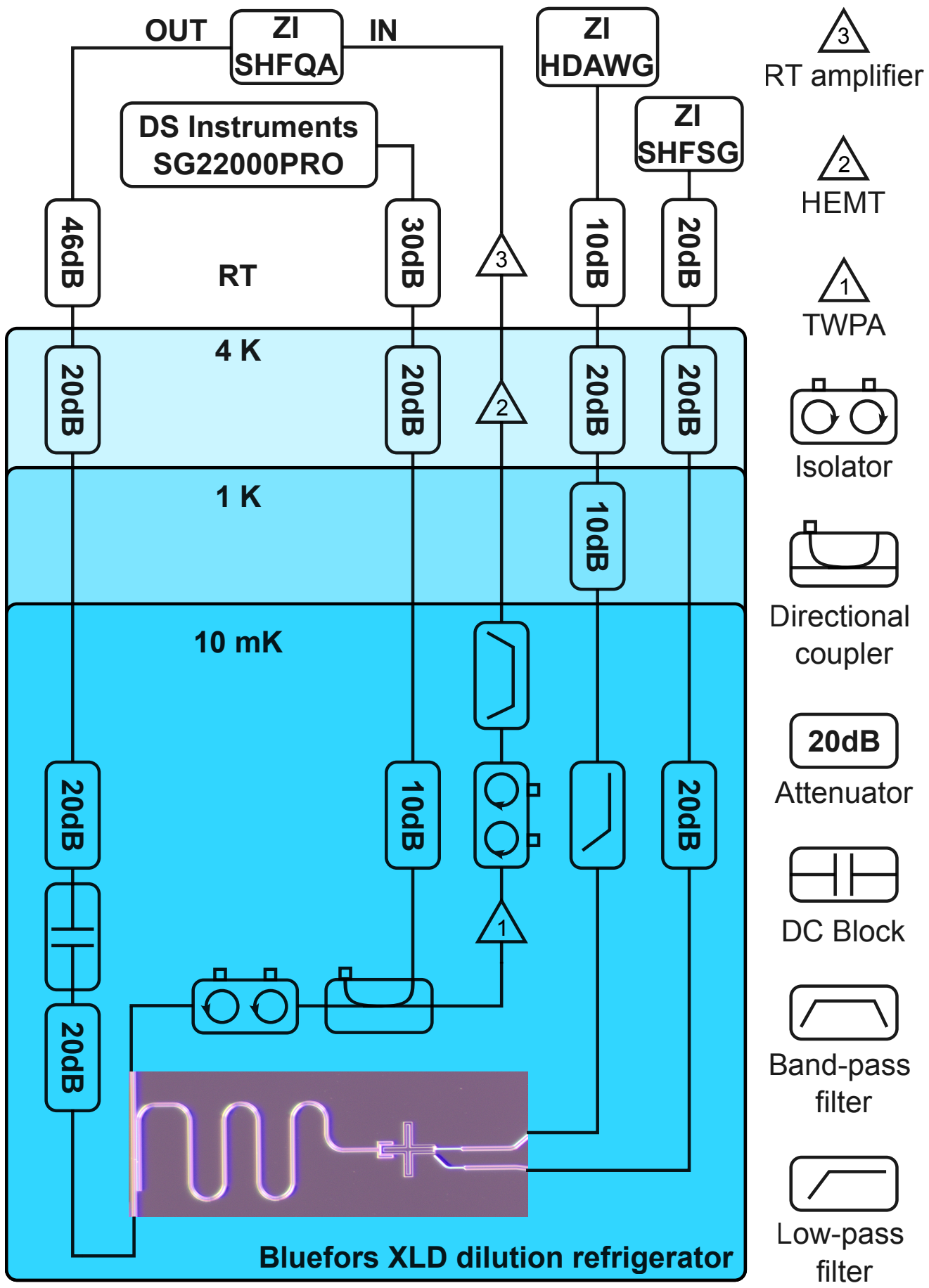

Supplementary Fig. 5. **The electronics and control wiring schematic.**

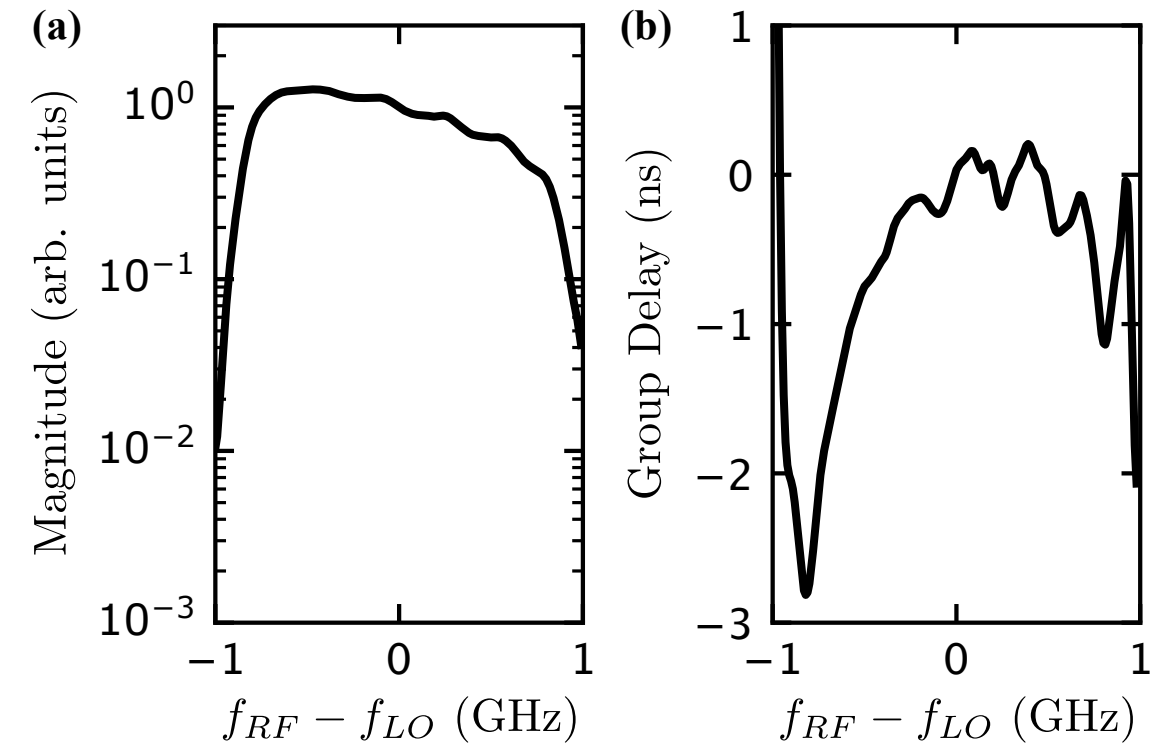

Supplementary Fig. 6. **The transfer function of the system.** (a) The magnitude of the transfer function. (b) The group delay of the transfer function.

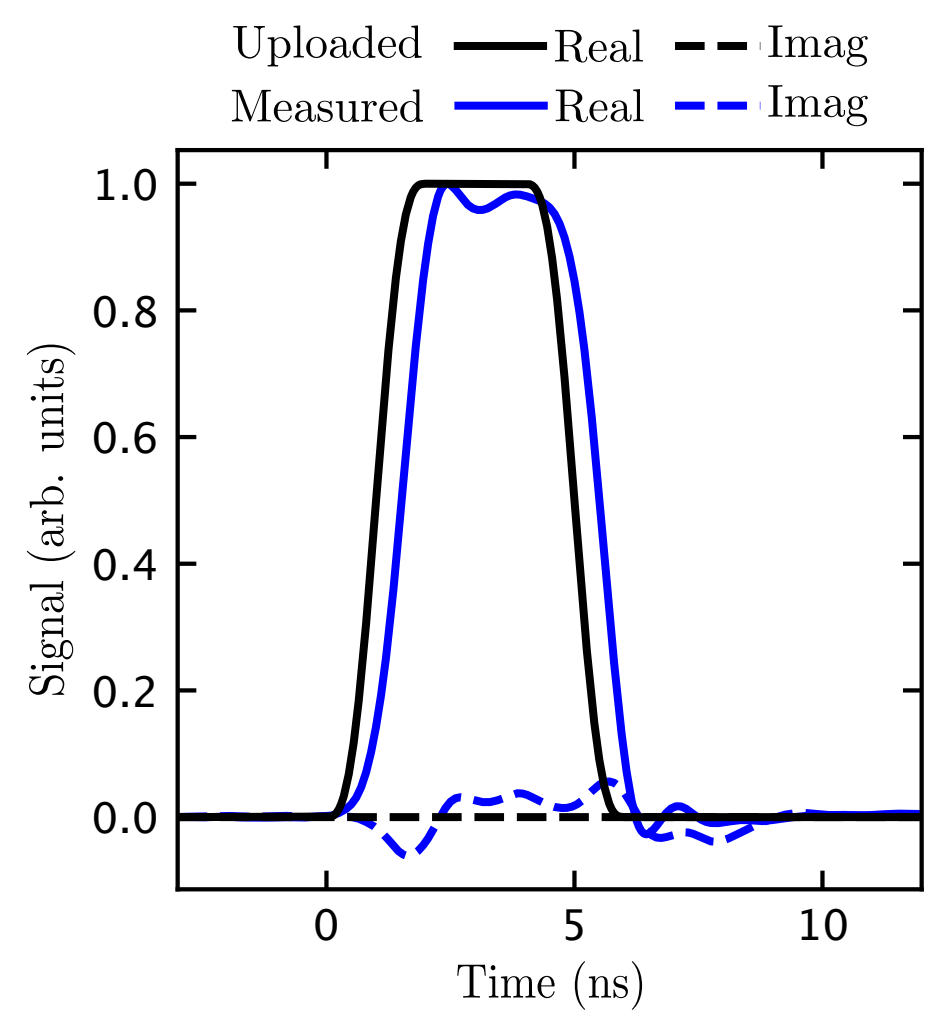

Supplementary Fig. 7. **The pulse envelopes under pulse distortion.** The data is measured with oscilloscope, and plotted after demodulation and square filtering with bandwidth ±1.5 GHz. The black lines are the pulse envelope uploaded to the waveform memory, and the blue lines are measured at the end of the wiring in the mixing chamber in the ambient condition. Both envelopes are normalized to the maximum of the absolute value.

tion for sub-10 ns pulse, even though it exceeds the instantaneous bandwidth based on the specification from Zurich Instruments. We further illustrate the effect of the pulse distortion in time domain as shown in Supplementary Fig. 7. We plot a flattop envelope of 6 ns with 2 ns ramp time measured from an oscilloscope connected to the wiring at the MXC. We note that the pulse distortion leads to a tail longer than few nanoseconds, which is detrimental to pulses duration we are investigating.

SUPPLEMENTARY NOTE 6: DERIVATION OF AMPLIFIED LEAKAGE ERROR

Here, we derive the amplification phases of amplified leakage error (ALE) sequence, and extract the leakage error.

In the absence of phase and amplitude errors, the unitary of an X operation with only leakage to $|2\rangle$ can be written as

$$X = \begin{pmatrix} 0 & 1 & A_1 e^{-i(\phi_1+\phi_2)} \\ 1 & 0 & A_0 e^{-i(\phi_0+\phi_2)} \\ -A_0 e^{i(\phi_0-\phi_2)} & -A_1 e^{i(\phi_1-\phi_2)} & e^{-i2\phi_2} \end{pmatrix}, \tag{9}$$

where $A_0, A_1 \ll 1$ are the leakage population to $|2\rangle$, ϕ_0, ϕ_1 are phases of the leakage population, and $2\phi_2$ is the phase acquired in $|2\rangle$ during X. The leakage error can be calculated as $\epsilon_{\text{leak},|2\rangle} = (A_0^2 + A_1^2)/2$. A virtual-Z rotation with angle θ can be written as

$$\text{VZ}(\theta) = \begin{pmatrix} 1 & 0 & 0 \\ 0 & e^{-i\theta} & 0 \\ 0 & 0 & e^{-i2\theta} \end{pmatrix}. \tag{10}$$

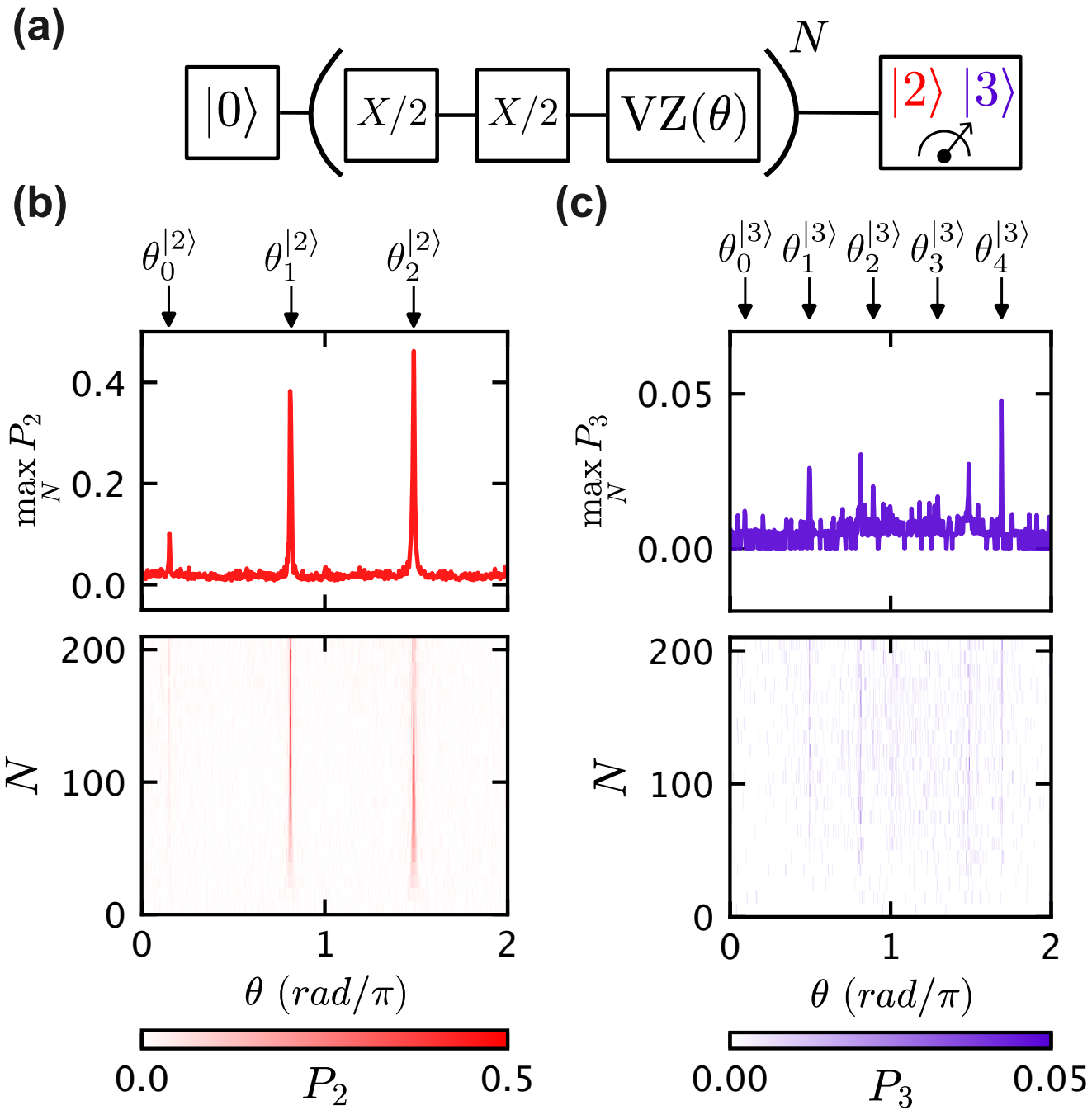

Supplementary Fig. 8. **ALE results for $X/2$ gates.** (a) The pulse sequence. (b) The $|2\rangle$ leakage population as a function of θ and N. The line plot on top shows the maximum leakage population for each θ. The amplification phases are at $\theta_k^{|2\rangle} = (0.153 + 2k/3)\pi,\ k = 0, 1, 2$. (c) Same as (b) but plotted with $|3\rangle$ leakage population. The amplification phases are at $\theta_k^{|3\rangle} = (0.09 + 2k/5)\pi,\ k = 0, 1, 2, 3, 4$.

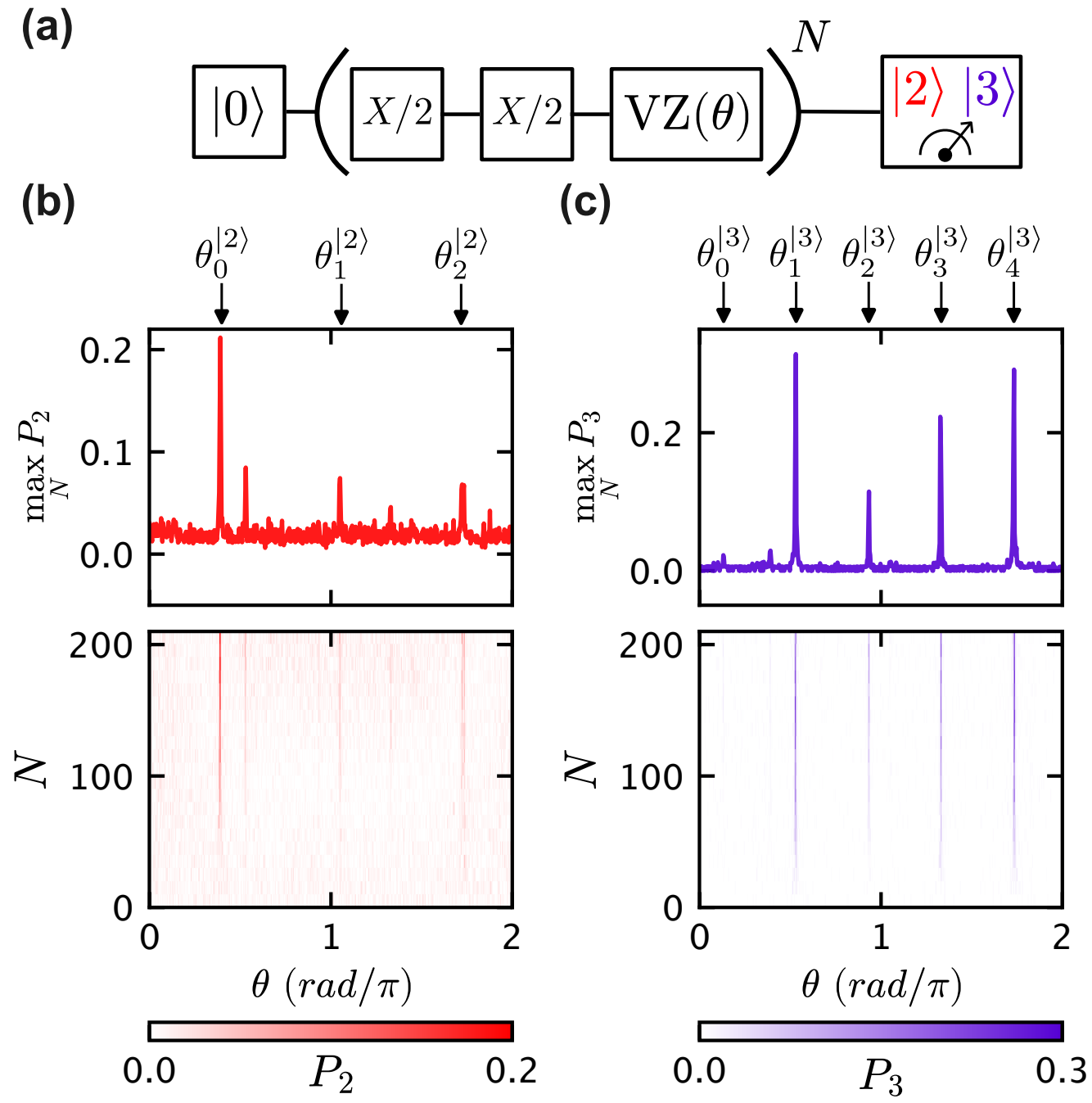

Supplementary Fig. 9. **ALE results for $X/2$ gates.** (a) The pulse sequence. (b) The $|2\rangle$ leakage population as a function of θ and N. The line plot on top shows the maximum leakage population for each θ. The amplification phases are at $\theta_k^{|2\rangle} = (0.387 + 2k/3)\pi,\ k = 0, 1, 2$. (c) Same as (b) but plotted with $|3\rangle$ leakage population. The amplification phases are at $\theta_k^{|3\rangle} = (0.13 + 2k/5)\pi,\ k = 0, 1, 2, 3, 4$.

Using Supplementary Eq. (9) and Supplementary Eq. (10), every double applications of X and VZ will result in,

$$U = (\mathrm{VZ}(\theta)\cdot X)^2 = \begin{pmatrix} 1 & 0 & u_{13} \\ 0 & 1 & u_{23} \\ u_{31} & u_{32} & e^{-i(4\phi_2+3\theta)} \end{pmatrix}. \tag{11}$$

with u_{ij} being complex elements of U. In order to coherently accumulate the leakage population, the amplification phases θ needs to satisfy

$$4\phi_2 + 3\theta_k = 2k\pi. \tag{12}$$

At the amplification phases θ_k, the leakage population can be coherently accumulated as it has the same phase after each operation. Meanwhile, the thermal excitation to $|2\rangle$ has different phases after each operation which leads to an incoherent accumulation. This indicates the ability to filter out the thermal excitation.

To extract the leakage rate, we first obtain the effective Hamiltonian of the system,

$$U^{N/2} = e^{iH_{\text{eff}}N/\hbar} \approx (I + i\frac{2}{\hbar}H_{\text{eff}})^{N/2}. \tag{13}$$

Then we solve the the time dependent Schrödinger equation using H_{eff},

$$-\frac{i}{\hbar}H_{\text{eff}}|\Psi(N)\rangle = \frac{d}{dN}|\Psi(N)\rangle, \tag{14}$$

with $|\Psi(0)\rangle = |0\rangle$, the $|2\rangle$ population after each operation can be expressed as,

$$P_2 = |\langle 2|\Psi(N)\rangle|^2 = a_k \sin^2(\omega_k N/2), \tag{15}$$
$$a_k\omega_k^2 = A_0^2 + A_1^2 - 2A_0A_1\cos(\Phi + \theta_k), \tag{16}$$

where N is the number of applications, $a_k = 1/2$ for $|2\rangle$, and Φ is a function of ϕ_0, ϕ_1, ϕ_2. Using Supplementary Eq. (16) and the identity $\sum_{k=1}^{n}\cos(a + 2k\pi/n) = 0$,

$$\epsilon_{\text{leak},|2\rangle} = \frac{1}{6}\sum_{k=1}^{3} a_k\omega_k^2. \tag{17}$$

In the case where $\omega_k N \ll 2\pi$, the Supplementary Eq. (15) reduces to $P_2 = a_k\omega_k^2 N^2/4$. This can be used as the fit model when the leakage is small.

The same analysis can be applied to $|3\rangle$. The last row of the X operation can be written as

$$x_{3,:}^T = \begin{pmatrix} -B_0 e^{i(\phi_0-\phi_3)} \\ -B_1 e^{i(\phi_1-\phi_3)} \\ -B_2 e^{i(\phi_2-\phi_3)} \\ e^{-i2\phi_3} \end{pmatrix}, \tag{18}$$

where B_i are the leakage population from $|i\rangle$ to $|3\rangle$. We then have the coherent accumulation condition as

$$4\phi_3 + 5\theta = 2k\pi, \tag{19}$$

and the leakage error at different amplification phases satisfies,

$$P_3 = a_k \sin^2(\omega_k N), \tag{20}$$

$$\epsilon_{\mathrm{leak},|3\rangle} = \frac{1}{10}\sum_{k=1}^{5} a_k \omega_k^2 = \frac{1}{2}(B_0^2 + B_1^2). \tag{21}$$

We would like to point out that the a_k for $|3\rangle$ depends on leakage rates B_2, whereas the final leakage error for the X operation does not. This indicates that ALE sequence is insensitive to transitions between $|2\rangle$ and $|3\rangle$.

Complementing the ALE results for X gates shown in Fig. 2 of the main text, we present two additional datasets of ALE results for $X/2$ gates in Supplementary Fig. 8 and Supplementary Fig. 9. In Supplementary Fig. 8(b), the three amplification phases are clearly visible, and they are evenly spaced in 2π period. In Supplementary Fig. 8(b), only two of five peaks $\theta_1^{|3\rangle}$ and $\theta_4^{|3\rangle}$ are clearly visible due to low leakage rate to $|3\rangle$. Supplementary Fig. 9 shows a different dataset for $X/2$ with different pulse parameters and duration, where the leakage to $|3\rangle$ is higher. In Supplementary Fig. 9(c), four of five peaks are clearly visible, the remaining one at $\theta_0^{|3\rangle}$ is only slightly above noise level, and the peaks are evenly spaced by $2\pi/5$.

SUPPLEMENTARY NOTE 7: AMPLIFIED PHASE ERROR FOR SINGLE QUBIT GATE

The amplified phase error(APE) is first introduced in Ref. [5], which allows to amplify a small phase error for $X/2$ gates. While Ref. [5] mentions that the protocol does not apply to X gates due to the cancellation of the phase error, there are multiple experimental implementations [2, 6, 7] shows the same protocol is applicable to X gates as well. Here, we provide a theoretical foundation for APE that applies to both X and $X/2$ gates.

Based on the fact that the phase error can be mitigated by introducing a constant detune, we assume the single qubit gate with phase errors can be decomposed as

$$X'_\theta = Y_{-\epsilon} X_\theta Y_\epsilon, \tag{22}$$

where θ is the rotation angle of the gate, and ϵ relates to the phase error of interest with $|\epsilon| \ll 1$. We note that Supplementary Eq. 22 corresponds to a rotation about a new axis ϵ away from the x axis in $X-Z$ plane, and it has the same form as the off-resonant drive. And we have

$$(X_\theta^\dagger)' = Y_\epsilon X_{-\theta} Y_{-\epsilon}, \tag{23}$$

because the inverse of a gate is realized through a π phase difference in the experimental implementation.

For an arbitrary rotation ϕ around the x or y axis, the gate operations are

$$X_\phi = \begin{pmatrix} \cos\phi/2 & -i\sin\phi/2 \\ -i\sin\phi/2 & \cos\phi/2 \end{pmatrix}, \tag{24}$$

$$Y_\phi = \begin{pmatrix} \cos\phi/2 & -\sin\phi/2 \\ \sin\phi/2 & \cos\phi/2 \end{pmatrix}. \tag{25}$$

Using APE protocol for $X/2$ gates, we have the pseudo-identity as

$$\begin{aligned} I'_{\pi/2} &= (X_{\pi/2}^\dagger)' X'_{\pi/2} \\ &= \frac{1}{2}\begin{pmatrix} 1+\cos 2\epsilon - 2i\sin\epsilon & -\sin 2\epsilon \\ \sin 2\epsilon & 1+\cos 2\epsilon + 2i\sin\epsilon \end{pmatrix} \\ &\approx \begin{pmatrix} 1-i\epsilon & -\epsilon \\ \epsilon & 1+i\epsilon \end{pmatrix}. \end{aligned} \tag{26}$$

This gives the same conclusion as in Ref. [5]. We note that the Supplementary Eq. 26 is actually a rotation of $2\sqrt{2}\epsilon$ around the axis $(z-y)/\sqrt{2}$ (Hadamard-like) in the case of $|\epsilon| \ll 1$, which explains well the experimental result in Supplementary Fig. 10(e).

Now we look at the APE protocol for X gates. Using APE protocol for X gates, we have the pseudoidentity as

$$\begin{aligned} I'_\pi &= (X_\pi^\dagger)' X'_\pi \\ &= \begin{pmatrix} \cos 2\epsilon & -\sin 2\epsilon \\ \sin 2\epsilon & \cos 2\epsilon \end{pmatrix}. \end{aligned} \tag{27}$$

We note that Supplementary Eq. 27 is actually a rotation of 4ϵ around the axis y, which explains well the experimental result in Supplementary Fig. 10(d). The Supplementary Eq. 27 shows that the APE protocol is applicable to X gates.

SUPPLEMENTARY NOTE 8: SINGLE QUBIT GATE CALIBRATION PROCEDURE

In this section, we give more details about the single qubit gate calibration procedure. We use the amplified amplitude error method (AAE) [8], also called ping-pong experiment, to minimize the amplitude error. We also use the amplified phase error approach (APE) [5] to minimize the phase error. In Supplementary Fig. 10, we show the schematic of the single qubit gate calibration procedure. We assume the qubit frequency f_q and anharmonicity Δ are well calibrated by Ramsey experiments before the Nelder-Mead (N-M) optimization. In Supplementary Fig. 10(a), we show the flow chart of the optimization procedure. For each iteration in N-M optimization, a set of $(\alpha_{12}, \alpha_{02}, \alpha_{13})$ is chosen by the optimizer. We first run

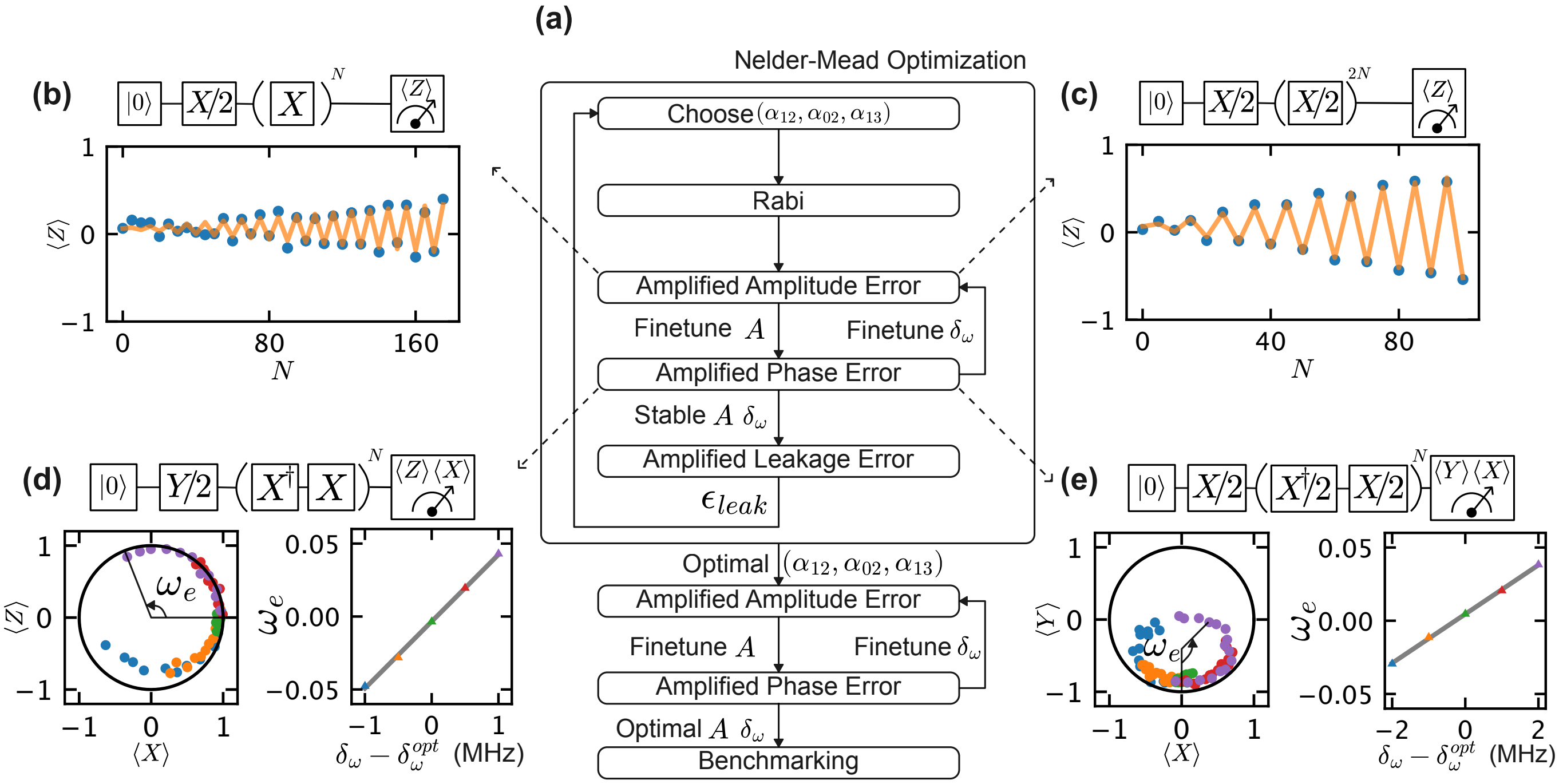

Supplementary Fig. 10. **Schematic of the single qubit gate calibration procedure.** (a) Flow chart of the Nelder-Mead optimization. We assume the qubit frequency f_q and the anharmonicity Δ is well calibrated before the Nelder-Mead optimization. (b)(c) The amplified amplitude error experiments for X gate (b) and for $X/2$ gate (c). The pulse sequence, the raw result (blue dots), and the fit (orange line) are shown. (d)(e) The amplified phase error experiments for X gate (d) and for $X/2$ gate (e). The pulse sequence, the raw result (colored symbols) and the fit (gray line) are shown.

a Rabi experiment to coarsely calibrate the pulse amplitude. Then, we use iterative AAE and APE experiments to finetune the pulse amplitude A and the constant detuning δ_ω. After both parameters are stable (typically we need two iterations), an ALE experiment is performed to evaluate the leakage error ϵ_{leak}. This ϵ_{leak} will be used as the loss function for optimizer to choose the next set of parameters until the algorithm converges. After the N-M optimization, extra iterations of AAE and APE are used to perform the final adjustments on the pulse amplitude A and the constant detuning δ_ω.

Supplementary Fig. 10(b)-(c) show examples of AAE experiments for both X and $X/2$ gates. The results are fitted to the function

$$f = a \sin\left(N(\pi + \phi_e)\right), \tag{28}$$

where ϕ_e is the over-/under-rotation angle.

Supplementary Fig. 10(d)-(e) show examples of APE experiments for both X and $X/2$ gates. Note the differences in the initial rotation and the final measurements. The error in the constant detuning can be extracted by fitting to the model

$$f = r_I \cos(\omega_e N + \phi_0) + i r_Q \sin(\omega_e N + \phi_0) + c_I + i c_Q, \tag{29}$$

where the rotation frequency ω_e is proportional to the errors in the constant detuning, and $\omega_e = 0$ when the constant detuning is optimal. Theoretically, the rotation radii $(r_I, r_Q) = (1, 1)$ for X gate and $(r_I, r_Q) = (1, 0.5)$ for $X/2$ gate, the initial angle $\phi_0 = 0$ for X gate and $\phi_0 = -\pi/2$ for $X/2$ gate, the rotation centers $(c_I, c_Q) = (0, 0)$ for X gate and $(c_I, c_Q) = (0, -0.5)$ for $X/2$ gate.

SUPPLEMENTARY NOTE 9: CLIFFORD-BASED RANDOMIZED BENCHMARKING

The comprehensive benchmarking of the single qubits gates is done through standard randomized benchmarking (RB) [9], leakage randomized benchmarking (LRB) [10], and purity benchmarking (PRB) [11]. We use RB to characterise the total gate error ϵ_{tot}, LRB to characterize leakage error ϵ_{leak}, and PRB to characterize the decoherence error ϵ_{decoh}. The benchmarkings are run in an interleaved fashion to obtain the error for the target gate. See Supplementary Fig. 11 for schematics of all benchmarking experiments.

All three benchmarking protocols include running multiple random sequences that consist of random Clifford gates C_i in different length (N) and a final recovering gate C_r.

RB measures the averaged population (P) of $|0\rangle$ at the end of each sequence, and fits it to model

$$P = A + Bp^N. \tag{30}$$

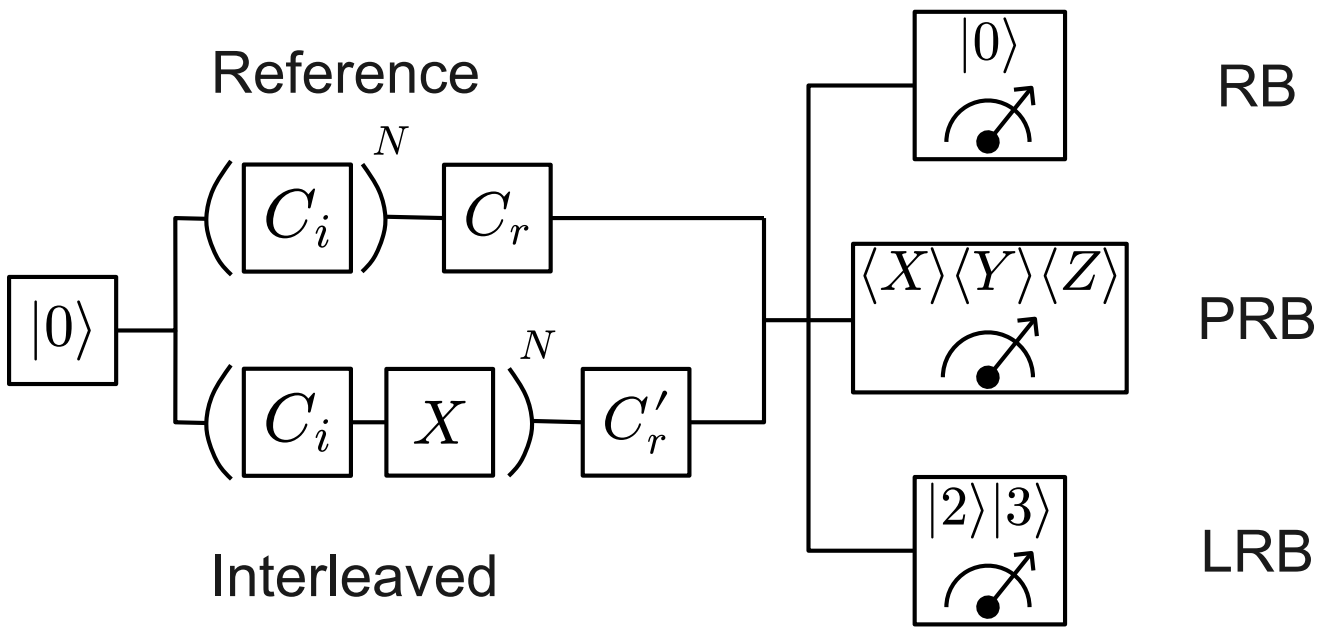

Supplementary Fig. 11. **Schematics of all benchmarking experiments.** Both reference and interleaved sequences are shown.

The total gate error of the target gate operation is calculated by

$$\epsilon_{tot} = (1 - p_{int}/p_{ref})/2, \tag{31}$$

where p_{ref} and p_{int} are fitted results from reference and interleaved sequences.

LRB measures the averaged population of leakage states, and fits it to model in Supplementary Eq. (30). The average leakage error of Clifford gates is $\epsilon = A(1-p)$, and the leakage error of the target gate operation is

$$\epsilon_{leak} = \epsilon_{int} - \epsilon_{ref}, \tag{32}$$

where ϵ_{ref} and ϵ_{int} are leakage results calculated from reference and interleaved sequences.

PRB measures the averaged purity $U = \langle X\rangle^2 + \langle Y\rangle^2 + \langle Z\rangle^2$ at the end of each sequence, fits it to model

$$U = A + Bu^N. \tag{33}$$

We use $X/2$ and $Y/2$ gate after each sequence to project the qubit state to different axes, and $\langle Z\rangle = P_0 - P_1$ without the normalization of $P_0 + P_1 = 1$. The purity error of the target gate operation is calculated by

$$\epsilon_{pur} = \left(1 - \sqrt{u_{ref}/u_{int}}\right)/2, \tag{34}$$

where u_{ref} and u_{int} are the fitted results from reference and interleaved sequences. As mentioned in [11], the decoherence error is obtained by $\epsilon_{decoh} \approx \epsilon_{pur} - \epsilon_{leak}/2$ in the presence of leakage channels.

For the Clifford gates decomposition, we follow the mapping stated in Ref. [12]. The $t_p = 8$ ns X gate is used when the pulse lengths of $X/2$ gate are swept, and vice versa. As the waveform memory of the control electronics is limited, the benchmarkings cannot reach the saturation values. We use $A = (\Lambda^{ps}_{01} + \Lambda^{ps}_{10})/2$ for RB fitting, where Λ^{ps}_{ij} are the elements in the assignment matrix. We use $A = 0$ for PRB fitting. In the case of low leakage population in LRB, we use $P = (1-p)AN$ as the fit model and assume $A = -B$. Supplementary Fig. 12 and

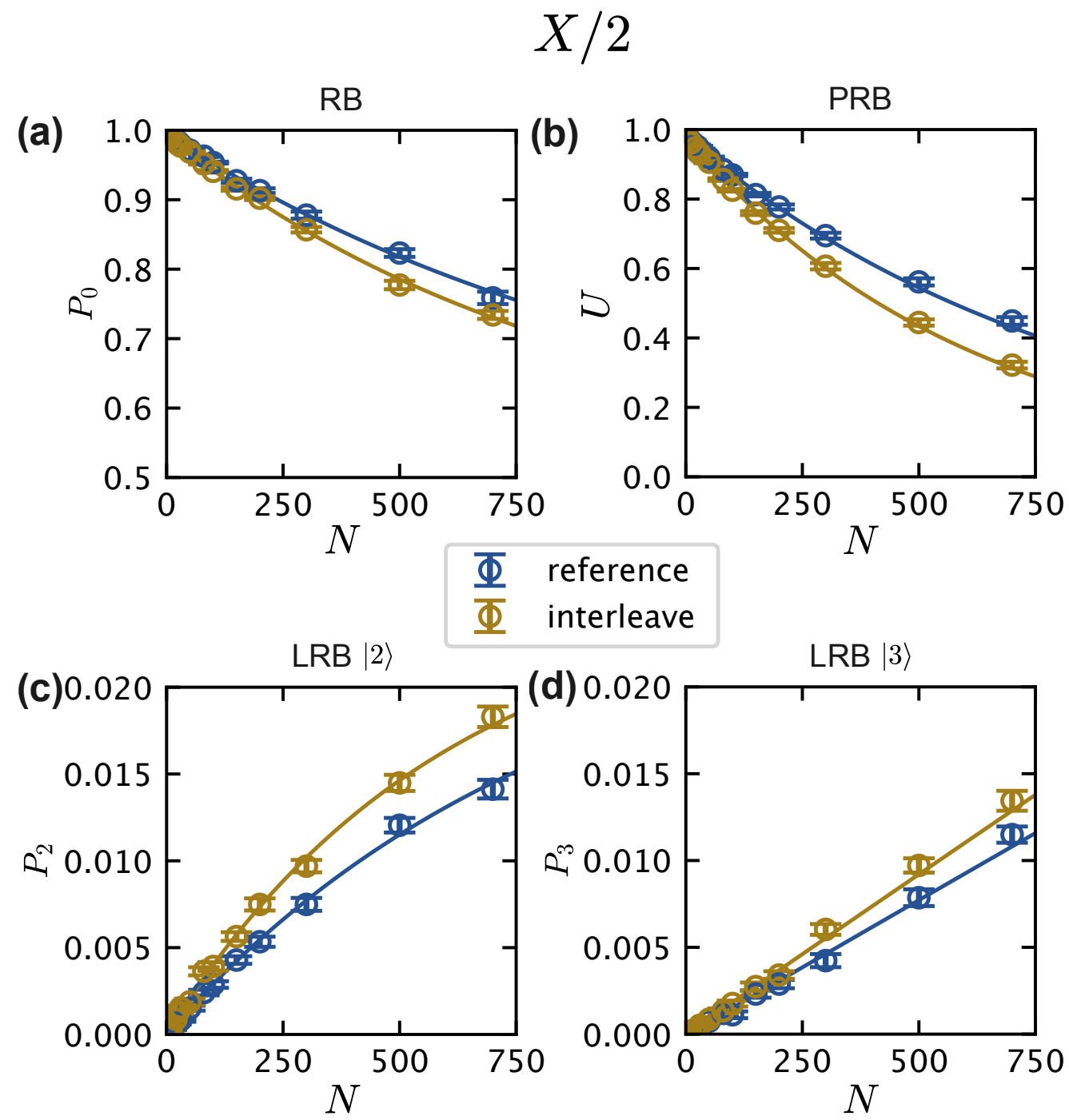

Supplementary Fig. 12. **Benchmarkings of the optimal $X/2$ gate at $t_p = 6.8$ ns, averaged over 60 random sequences.** The error bar indicates the standard error of the mean. The solid lines are fit curves. (a) RB. (b) PRB. (c) LRB for $|2\rangle$. (d) LRB for $|3\rangle$.

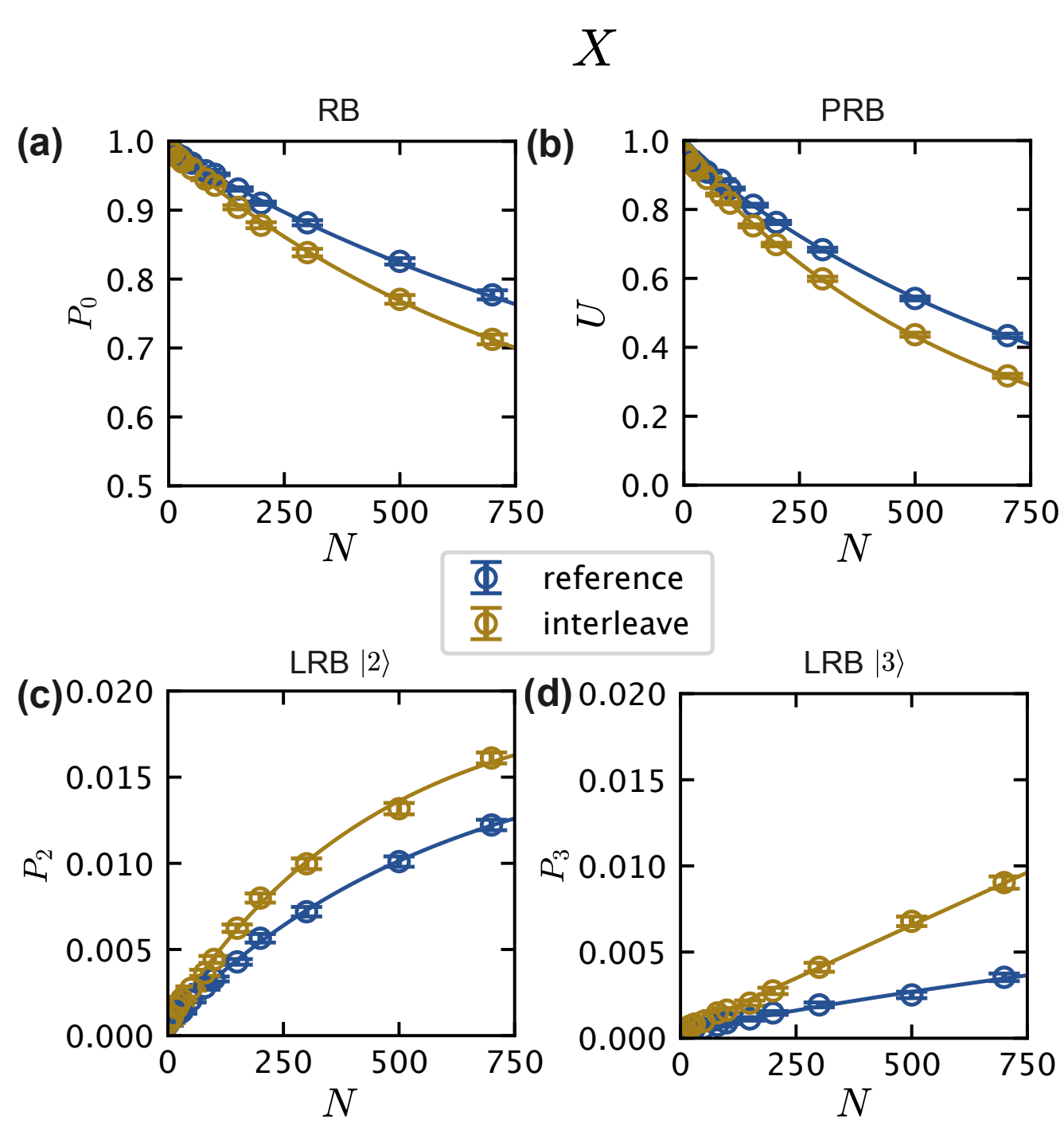

Supplementary Fig. 13. **Benchmarkings of the optimal X gate at $t_p = 6.8$ ns, averaged over 60 random sequences.** The error bar indicates the standard error of the mean. The solid lines are fit curves. (a) RB. (b) PRB. (c) LRB for $|2\rangle$. (d) LRB for $|3\rangle$.

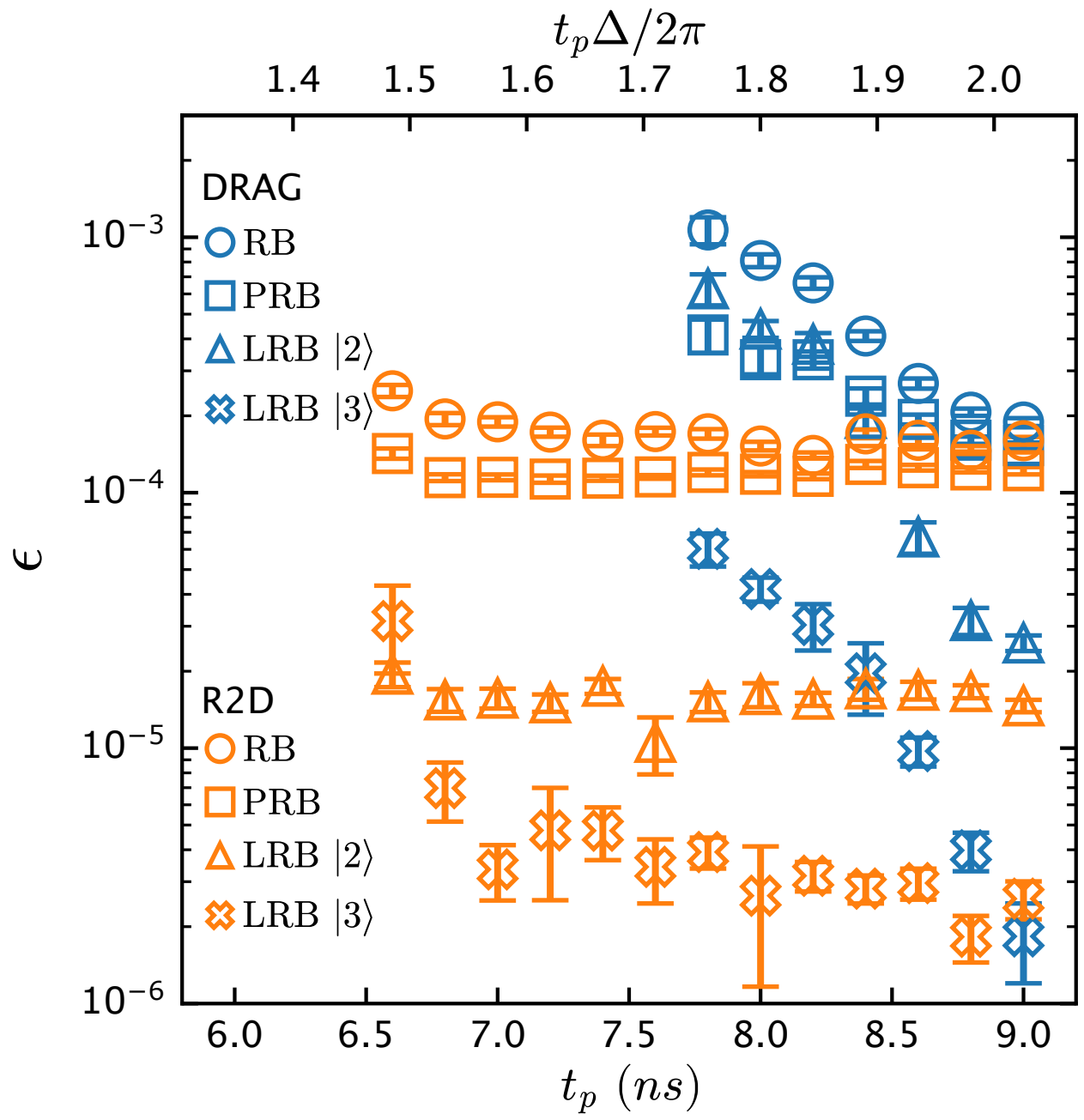

Supplementary Fig. 14. **Benchmarking results for X gates using the standard DRAG and the R2D methods.** R2D outperforms standard DRAG. Error bars are the standard error from the fit.

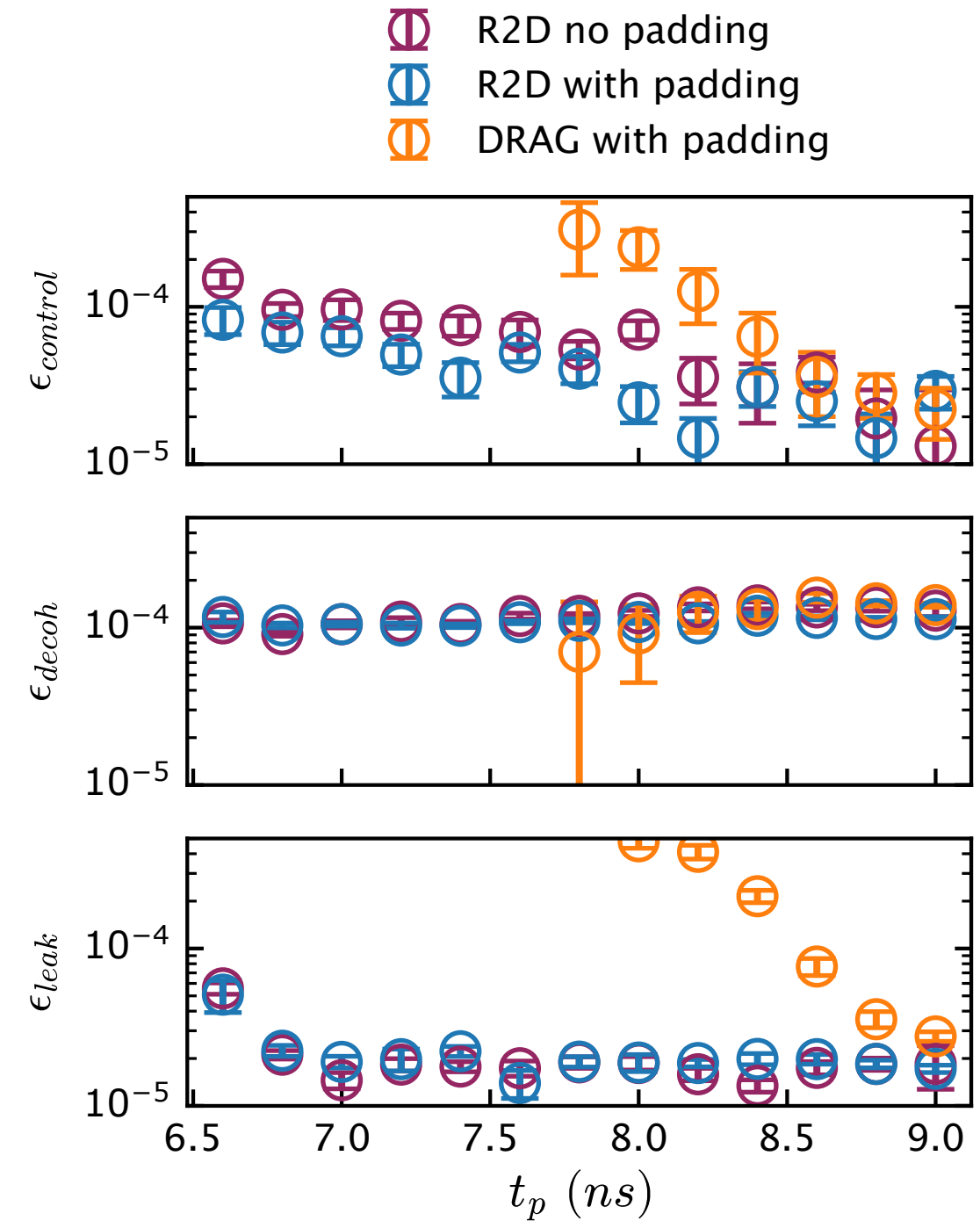

Supplementary Fig. 15. **The error budgets for the R2D and DRAG pulse shapes.** The padding reduces control error (top) by $1 \sim 4 \times 10^{-5}$, and does not influence the decoherence error (middle) and the leakage error (bottom). Error bars are the standard error from the fit.

Supplementary Fig. 13, we show the benchmark curves for the optimal $X/2$ and X gates at $t_p = 6.8$ ns, respectively. All benchmark curves are averaged over 60 random sequences.

We directly compare the benchmarking results for X gates using standard DRAG and R2D corrections in Supplementary Fig. 14. For the standard DRAG correction, the α_{12} is optimized to minimize the total leakage error, and α_{02} and α_{13} are set to 0.

SUPPLEMENTARY NOTE 10: CONTROL ERROR AND PULSE PADDING

Due to the presence of the microwave pulse distortion, we have included 1 ns zero padding after the pulse. To further elucidate the effect of the padding, we have run the same benchmarking for pulses without padding.

In Supplementary Fig. 15, we compare the error budgets between different pulse configurations. Comparing the curves R2D with padding (blue) and DRAG with padding (orange), we see that introducing R2D pulse shaping does not increase the control error, but rather decreases it. In addition, the correlated increasing trend of control and leakage error observed when using DRAG, which is absent when using R2D, suggests that the reduction in leakage achieved by introducing R2D improves the calibration accuracy.

Supplementary Fig. 15 also shows that the padding after the pulse duration effectively decreases the control error while showing no influence on decoherence error and leakage error (blue and purple). The control error decreases around $1 \sim 4 \times 10^{-5}$ with 1 ns padding after the pulse. This feature matches well with the notion that control error is caused by pulse distortions. Moreover, if the additional control error is a consequence of the R2D pulse shape, such error would be confined within the pulse duration and should not be affected by the padding after the pulse.

[1] J. D. D. C. Jesus, B. Li, Y. Gao, R. Barends, F. A. Cárdenas-López, and F. Motzoi, Analytical blueprint for 99.999% fidelity x-gates on present superconducting hardware under strong driving, PRX Quantum **7**, 10.1103/sdxb-v39h (2026).

[2] Z. Chen, J. Kelly, C. Quintana, R. Barends, B. Campbell, Y. Chen, B. Chiaro, A. Dunsworth, A. Fowler, E. Lucero, E. Jeffrey, A. Megrant, J. Mutus, M. Neeley, C. Neill, P. O'Malley, P. Roushan, D. Sank, A. Vainsencher, J. Wenner, T. White, A. Korotkov, and J. M. Martinis, Measuring and suppressing quantum state leakage in a superconducting qubit, Physical Review Letters **116**, 10.1103/physrevlett.116.020501 (2016).

[3] D. Zeuch *et al.*, Exact rotating wave approximation, Annals of Physics **423**, 168327 (2020).

[4] N. Lambert, E. Giguère, P. Menczel, B. Li, P. Hopf,

G. Suárez, M. Gali, J. Lishman, R. Gadhvi, R. Agarwal, A. Galicia, N. Shammah, P. Nation, J. Johansson, S. Ahmed, S. Cross, A. Pitchford, and F. Nori, Qutip 5: The quantum toolbox in python, Physics Reports **1153**, 1 (2026).

[5] E. Lucero, J. Kelly, R. C. Bialczak, M. Lenander, M. Mariantoni, M. Neeley, A. D. O'Connell, D. Sank, H. Wang, M. Weides, J. Wenner, T. Yamamoto, A. N. Cleland, and J. M. Martinis, Reduced phase error through optimized control of a superconducting qubit, Physical Review A **82**, 10.1103/PhysRevA.82.042339 (2010).

[6] L. Ding, M. Hays, Y. Sung, B. Kannan, J. An, A. Di Paolo, A. H. Karamlou, T. M. Hazard, K. Azar, D. K. Kim, B. M. Niedzielski, A. Melville, M. E. Schwartz, J. L. Yoder, T. P. Orlando, S. Gustavsson, J. A. Grover, K. Serniak, and W. D. Oliver, High-fidelity, frequency-flexible two-qubit fluxonium gates with a transmon coupler, Phys. Rev. X **13**, 031035 (2023).

[7] Z. Li, P. Liu, P. Zhao, Z. Mi, H. Xu, X. Liang, T. Su, W. Sun, G. Xue, J.-N. Zhang, W. Liu, Y. Jin, and H. Yu, Error per single-qubit gate below 104 in a superconducting qubit, npj Quantum Information **9**, 10.1038/s41534-023-00781-x (2023).

[8] S. Sheldon, L. S. Bishop, E. Magesan, S. Filipp, J. M. Chow, and J. M. Gambetta, Characterizing errors on qubit operations via iterative randomized benchmarking, Physical Review A **93**, 10.1103/physreva.93.012301 (2016).

[9] E. Magesan, J. M. Gambetta, and J. Emerson, Scalable and robust randomized benchmarking of quantum processes, Physical Review Letters **106**, 10.1103/physrevlett.106.180504 (2011).

[10] C. J. Wood and J. M. Gambetta, Quantification and characterization of leakage errors, Physical Review A **97**, 10.1103/physreva.97.032306 (2018).

[11] J. Wallman, C. Granade, R. Harper, and S. T. Flammia, Estimating the coherence of noise, New Journal of Physics **17**, 113020 (2015).

[12] R. Barends, J. Kelly, A. Veitia, A. Megrant, A. G. Fowler, B. Campbell, Y. Chen, Z. Chen, B. Chiaro, A. Dunsworth, I.-C. Hoi, E. Jeffrey, C. Neill, P. J. J. O'Malley, J. Mutus, C. Quintana, P. Roushan, D. Sank, J. Wenner, T. C. White, A. N. Korotkov, A. N. Cleland, and J. M. Martinis, Rolling quantum dice with a superconducting qubit, Phys. Rev. A **90**, 030303 (2014).
