# Supplementary material for: Ultrafast single-qubit gates through multi-photon transition removal
Source: Nat Commun. 2026 Oct 23;17:10874. doi: 10.1038/s41467-026-78373-4 (PMC13694273; doi:10.1038/s41467-026-78373-4)
Supplement: Supplementary file 2 [file 41467_2026_78373_MOESM2_ESM.pdf]

nature portfolio

Peer Review File

Ultrafast Single Qubit Gates through Multi-Photon Transition Removal

Corresponding Author: Mr Yuan Gao

This file contains all reviewer reports in order by version, followed by all author rebuttals in order by version.

Attachments originally included by the reviewers as part of their assessment can be found at the end of this file.

Version 0:

Reviewer comments:

Reviewer #1

(Remarks to the Author)
See the attached pdf file.

Reviewer #2

(Remarks to the Author)
Review NCOMMS-25-100659-T

The manuscript "Ultrafast single qubit gates through multi-photon transition removal" has been submitted for consideration in Nature Communications. The authors present a modified implementation of the DRAG technique aimed at further suppressing leakage outside the computational subspace. They report leakage errors below 2 10e-5 and introduce a protocol to amplify leakage errors, enabling their characterization at the 10e-6 level.

Despite these technically sound results, we do not consider the manuscript to meet the criteria for publication in Nature Communications. While the experimental performance is solid, the underlying concept builds directly on the well-established DRAG framework. The extension of DRAG to include higher energy levels, leading to incremental improvements in leakage suppression, represents a natural and expected generalization rather than a conceptual advance. As such, the work does not, in our view, rise to the level of important advances required by Nature Communications.

Below, we provide several comments that we believe will assist the authors in any future revision process

- We find that much information is missing from the manuscript; the reference points to a manuscript (J. D. Da Costa Jesus et al. (2025) that is still in preparation. This makes it impossible to judge some of the concepts and equations presented in this work. We suggest that the authors add part of the findings to the supplementary material.

-Waveform Limits and Predistortion
- The 9ns limit for waveform memory feels quite tight.
- The authors mention in the supplement that distortion correction is the reason for this time limit, but there is no data to back this up.
- Was the distortion measure at cryogenic temperature? How?
- It would be helpful to see a plot of fidelity with vs. without distortion correction. Even with 1ns of padding, it's hard to see why predistortion is so critical here without a visual comparison

- Unexpected Leakage at 6.6ns
- We noticed that at 6.6ns, leakage to the state |3> jumps by an order of magnitude. This is surprising because, according to Equation 2, the transition 1->3 should be much weaker than the 0->2 transition. What is driving this?
- Also, how are the solid lines (red/purple) being extracted? If these are simulations at optimal points satisfied by equations 5 and 6, shouldn't coherent errors theoretically be zero? If they aren't, which specific transitions are the main culprits?

- APE Protocol Implementation
In the calibration protocol, the authors have used the amplifying phase error (APE) technique, and cited E. Lucero et al. “Reduced phase error through optimized control of a superconducting qubit”, Physical Review A 82, 10.1103/PhysRevA.82.042339, (2010). After equation 3, the paper mentions that pi pulses cannot amplify error, as the phase error cancels. However, the authors have used this protocol, have they modified the protocol?

- In the conclusions, the authors mention that R2D helps with driving qubits simultaneously. Could they elaborate more on this? It’s not clear whether they refer to general crosstalk or specific resonant transitions triggered by the drive on Qubit A affecting Qubit B?

Reviewer #3

(Remarks to the Author)
I co-reviewed this manuscript with one of the reviewers who provided the listed reports. This is part of the Nature Communications initiative to facilitate training in peer review and to provide appropriate recognition for Early Career Researchers who co-review manuscripts.

Reviewer #4

(Remarks to the Author)
- What are the noteworthy results?

The paper by Y. Gao et al demonstrated a new pulse shaping technique for single qubit gates to suppress leakage and showcased very low leakage (below 2e-5) for 6.8 ns pi and pi/2 gate; the authors achieve such low leakage with a record value for the ratio of drive strength over qubit anharmonicity.

The paper also demonstrated Amplified Leakage Error (ALE) sequence that was used for both coherent leakage characterization and pulse parameter optimization; the technique was shown to be able to detect leakage as low as 1e-6.

- Will the work be of significance to the field and related fields? How does it compare to the established literature? If the work is not original, please provide relevant references.

Yes, the work is of significance to the field of superconducting qubits and superconducting quantum computing with a few caveats.

In terms of gate performance, for pi gates, the leakage number of 2e-5 for 6.8 ns gate is probably indeed the record for the same ratio of drive strength over qubit anharmonicity, as far as I know. However, experimental data in the paper shows significant increase in additional control error [Fig. 4c] as we go to short gates, which the authors attributed to pulse distortion. It is unclear how well we can mitigate this effect and it makes the practical usefulness of the pulse shaping technique questionable, at least for pi pulse.

For pi/2 gate, the leakage number reported in the paper is on a par with previous work published in PRX Quantum 5, 030353 which achieved 2e-5 leakage for 6.25 ns gate and even smaller anharmonicity.

Regarding the ALE sequence used for leakage characterization in this paper, I would argue that this is not new. A similar technique was used in Phys. Rev. Lett. 100, 247001 for characterizing coherent leakage in pi pulse, dubbed as “ramsey error filter”; recently it was generalized to pi/2 pulse in Phys. Rev. Lett. 135, 130601. The pulse sequence is exactly the same as used in the paper by Y. Gao et al. The only difference is that Y. Gao et al used virtual Z gate to accumulate phase whereas the aforementioned two papers add wait time after the gate. In fact it was mentioned in Phys. Rev. Lett. 135, 130601 that virtual Z gate and adding physical wait time achieve exactly the same effect.

With these said, I do believe the results of the paper, in particular, the pulse shaping technique is still of significance to the field. However, given the caveats I listed above, I do not think the paper suits a prestigious article as Nature Communications.

- Does the work support the conclusions and claims, or is additional evidence needed?

Yes, the work supports the conclusions and claims.

- Are there any flaws in the data analysis, interpretation and conclusions? - Do these prohibit publication or require revision?

There are a few places in the paper that require clarification and minor revision.

1) Expressions for lambda’s need to be given

2) It would be useful to comment why we need the fourth derivative in Eq. (3), that is, would we achieve similar performance

by setting alpha_02 or alpha_13 to zero.

3) The matrix for X in Eq. (7) does not appear to be unitary. Can the authors comment on why?

- Is the methodology sound? Does the work meet the expected standards in your field?

Yes and yes

- Is there enough detail provided in the methods for the work to be reproduced?

Yes

Version 1:

Reviewer comments:

Reviewer #1

(Remarks to the Author)
See the attached pdf file.

Reviewer #2

(Remarks to the Author)
After careful consideration, we remain unconvinced that this manuscript meets the level of novelty and impact expected for publication in a high-profile journal such as Nature Communications. We would therefore recommend transfer to NPJ Quantum Information or consideration for publication in other specialized journals, such as PRX Quantum.
While we appreciate the authors' approach and acknowledge the arguments presented, we do not consider the underlying concepts to be sufficiently novel. In particular, the idea of employing pulse shapes involving multiple derivatives to suppress undesired transitions has previously been introduced by Prof. F. Motzoi and collaborators in the context of the cross-resonance gate (Box Li at al, npj Quantum Information volume 10, Article number: 66 (2024)). The recursive extension of the DRAG formalism proposed in the submitted manuscript, aimed at simultaneously suppressing multiple leakage and error transitions, appears conceptually very similar to the methodology developed in that earlier work.
Although the manuscript provides a useful generalization and demonstrates the applicability of the approach in a different setting, we do not believe that the conceptual advance is substantial enough to justify publication in Nature Communications.

Reviewer #3

(Remarks to the Author)
I co-reviewed this manuscript with one of the reviewers who provided the listed reports. This is part of the Nature Communications initiative to facilitate training in peer review and to provide appropriate recognition for Early Career Researchers who co-review manuscripts.

Reviewer #4

(Remarks to the Author)
I do not find the responses from the authors completely satisfactory.

1) Regarding the practical usefulness of the technique, as shown in Fig. 4c), due to the increase in the control error, the general trend is that total gate error increases as gate duration becomes smaller. Because of this, it is unclear how practically useful it is to have single qubit gates with duration below 10 ns.

The authors emphasize that the goal of the technique is to suppress leakage error, mitigation of pulse distortion is beyond the scope of the work. I do not find this answer fully satisfactory because it is not very clear where exactly the additional control error is coming from. There is a possibility that the additional control error is a consequence of applying the R2D pulse.

2) Regarding the similarity between the ALE technique used by the authors and the technique used in PRL 100, 247001 and PRL 135, 130601 (2025), I also do not find the authors' response completely satisfactory. The authors stated that they observed three distinct amplification phases while PRL 135, 130601 only predicted two. The authors were probably referring to their results for pi pulse in Fig. 2b which show three distinct amplification phases. However, results in PRL 135, 130601 (2025) were for pi/2 pulses. They do not necessarily have the same number of amplification peaks as for pi pulse. It would

be more convincing if authors also show ALE results for pi/2 pulses which I do not find in the current manuscript.

Version 2:

Reviewer comments:

Reviewer #4

(Remarks to the Author)
The authors have addressed my comments. In my opinion, the manuscript is now suitable as a publication in the Nature Communications. One very minor comment is that in the captions of Fig. S8 and S9, I suggest the authors to write k = 0, 1, 2 instead of k = 0...2, and similarly write k = 0, 1, 2, 3, 4 instead of k = 0...4.

Open Access This Peer Review File is licensed under a Creative Commons Attribution 4.0 International License, which permits use, sharing, adaptation, distribution and reproduction in any medium or format, as long as you give appropriate credit to the original author(s) and the source, provide a link to the Creative Commons license, and indicate if changes were made.
In cases where reviewers are anonymous, credit should be given to 'Anonymous Referee' and the source.
The images or other third party material in this Peer Review File are included in the article's Creative Commons license, unless indicated otherwise in a credit line to the material. If material is not included in the article's Creative Commons license and your intended use is not permitted by statutory regulation or exceeds the permitted use, you will need to obtain permission directly from the copyright holder.
To view a copy of this license, visit https://creativecommons.org/licenses/by/4.0/

REPLY TO REVIEWERS

Reviewer #1:

In this paper, the authors experimentally demonstrated ultrafast and high-fidelity single qubit gates by using R2D method. The authors also introduced a method that can precisely quantify leakage rates. Improving gate operation is very important, and the present work addresses this relevant and timely topic. The measurements appear to have been carried out carefully, and the quality of the data is good enough.

In my view, the paper has the potential to meet the publication criteria of Nature communications.

We sincerely thank the reviewer for their assessments.

Fig. 1(b): "vertical solid line" would be better for the unwanted transition frequencies.

We have reworded the caption for Fig. 1(b).

What are the three lines in Fig. 3(e)? Is one of the curves in Fig. 3(e) cross section of the plot in Fig. 2(d)? What are the three lines in Fig. 3(f)? Is one of the curves in Fig. 3(f) cross section of the plot in Fig. 2(e)? The relation between them should be clarified.

We would like to thank the reviewer for pointing this out.

Fig. 2 and Fig. 3(e)(f) are from the same experiment, but different datasets. As shown in Fig. 2(b)(d), there are three distinct phases that amplify the leakage error to state $|2\rangle$. The three lines in Fig. 3(e) are the cross sections at these amplification phases. All three lines are necessary to extract the leakage error per gate. Fig. 2(c)(e) shows that there are five distinct phases that amplify the leakage error to state $|3\rangle$. And the cross sections at those amplification phases are plotted in Fig. 3(f). All five lines are necessary to extract the leakage error per gate.

We have updated the caption of Fig. 3(e)(f) to better explain their relation.

lines 241-244: The authors attribute the difference in leakage errors to the fact that the ALE method is insensitive to thermal excitations and $|2\rangle \rightarrow |3\rangle$ transition errors. The authors have to describe the reasons by referring Figures or equations.

We thank the reviewer for raising this point. As discussed in the section "Derivation of Amplified Leakage Error" in the supplementary material, the thermal excitations after each gate operation differ in phase, which leads to an incoherent accumulation. The Eq. S19 indicates that the extracted leakage error to $|3\rangle$ does not depend on leakage rate B_2 from $|2\rangle$ to $|3\rangle$. We have added a reference to the supplementary material as well as more explanations in the supplementary material.

Reviewer #2:

The manuscript "Ultrafast single qubit gates through multi-photon transition removal" has been submitted for consideration in Nature Communications. The authors present a modified implementation of the DRAG technique aimed at further suppressing leakage outside the computational subspace. They report leakage errors below 2 10e-5 and introduce a protocol to amplify leakage errors, enabling their characterization at the 10e-6 level.

Despite these technically sound results, we do not consider the manuscript to meet the criteria for publication in Nature Communications. While the experimental performance is solid, the underlying concept builds directly on the well-established DRAG framework. The extension of DRAG to include higher energy levels, leading to incremental improvements in leakage suppression, represents a natural and expected generalization rather than a conceptual advance. As such, the work does not, in our view, rise to the level of important advances required by Nature Communications.

We respectfully disagree with reviewer. Since Phys. Rev. Lett. 103, 110501(2009) showed the potential to suppress leakage while increasing the driving strength using the DRAG framework, many research groups have utilized DRAG

and its variants to push the gate performance further. Yet, after over a decade, the field has not fully recognized the fundamental cause of the remaining leakage in presence of DRAG correction in the strongly driven regime. In fact, the community exhaustively engineers the pulse spectrum using brute force by nulling larger frequency bandwidth, fails to consider transitions outside the nearest-neighbor levels, and handles the qubit under strong driving in the same manner as under weak perturbation. This can be clearly observed even in recent publications, PRX Quantum 5, 030353(2024), Phys. Rev. Lett. 135, 130601(2025).

This is understandable, as shown in Phys. Rev. A. 76, 042319(2007), the two-photon transition matrix elements in the transmon are negligible in the small perturbation limit. However this is no longer valid if the driving strength is large enough to enter the strongly driven regime.

Our work departs from standard heuristics about nearest-neighbor transitions and explicitly emphasizes the significant impact of multi-photon transitions in such context. Such transitions do not appear as a single frequency in pulse (Ω) spectrum, but rather in the spectrum of the pulse squared (Ω^2). This is also the first time ever that the spectrum of Ω^2 has been used for pulse shaping analysis. This novel insight about the existence of multi-photon transitions enables the achievements in this manuscript, and the effective suppression of leakage by addressing these transitions provides a clear evidence that such persistently overlooked transitions are the key factor in further advancing the field.

Importantly, theoretical proposals addressing multi-photon transitions are relatively few, not to mention their experimental realizations. Our work, as the first experimental implementation of multi-photon transition suppression during the gate operations, validates that the gate performance can be pushed even further with a straightforward pulse shaping informed by novel insights. In fact, the novelty extends far beyond superconducting qubits, since multi-photon transitions despite being ubiquitous in quantum dynamics have not previously been analytically canceled in any setting. Therefore, our work surpasses extending DRAG with incremental improvements, but instead gives a novel insight and arguably steers the research into a new direction in this field.

In summary, we strongly believe that the identification of the critical role of multi-photon transitions, as well as their suppression, in our work both elucidates the physics of strong driving, a foundational topic in qubit control that has thus far been ignored, and uses this to establish a novel platform for quantum control, enabling a record gate fidelity. This is in stark contrast to heuristic modifications of the pulse spectrum, that provide neither insight into the physics nor a pathway to scalable implementation. Therefore, we believe that the results presented in this manuscript will have a critical and profound impact on the field.

Below, we provide several comments that we believe will assist the authors in any future revision process

We find that much information is missing from the manuscript; the reference points to a manuscript (J. D. Da Costa Jesus et al. (2025) that is still in preparation. This makes it impossible to judge some of the concepts and equations presented in this work. We suggest that the authors add part of the findings to the supplementary material.

We thank the reviewer for the comments and valuable suggestions. The mentioned manuscript is now public in arXiv "arXiv:2512.19919", we have updated the reference. While the other manuscript "arXiv:2512.19919" focuses on the theoretical framework, our manuscript focuses on the experimental results.

-Waveform Limits and Predistortion

The 9ns limit for waveform memory feels quite tight.

The authors mention in the supplement that distortion correction is the reason for this time limit, but there is no data to back this up.

Was the distortion measure at cryogenic temperature? How?

We thank the reviewer for the questions.

The time limitation is caused by the combination of the limited waveform memory in the electronics and the way we apply the distortion correction.

We first measure the system transfer function from the electronics to the mixing chamber (MXC) of the fridge while the setup is in ambient condition. Then we apply the inverse of the transfer function to the sampled waveform, and upload the full waveform of the experiment to the waveform memory in the electronics. We did not perform

any in-situ distortion calibration. Uploading the full waveform strongly limits the number of gates we could execute during randomized benchmarking, thus restricts the benchmarking for longer gate durations or gate sequences.

The SHFSG in our setup has waveform memory of 96kSa and 2GSa/s. As shown in Fig. S8 and S9, 700 Clifford gates are required to extract gate error with reasonable confidence. In the worst case of randomized benchmarking, a single Clifford gate is decomposed into three single qubit gates. The reference and interleaved sequences are preferably run in the same experiment to have a better consistency. Therefore, for 10 ns pulse length (9 ns gate duration + 1 ns padding) a single randomized benchmaring sequence has $700 \times 3(\text{reference}) + 700 \times (3+1)(\text{interleaved}) = 4900$ gates $= 49\mu s = 98$ kSa. This fits into the waveform memory as long as we do not face the worst case.

Nevertheless, the manuscript deals with ultrafast single qubit gate, and we have compared with standard DRAG correction in Fig. S10. Pulses longer than 9 ns do not yield additional information. We have added more information about the distortion correction in the section "Experimental Setup and Signal Distortion" in the supplementary material.

It would be helpful to see a plot of fidelity with vs. without distortion correction. Even with 1ns of padding, it's hard to see why predistortion is so critical here without a visual comparison

We thank the reviewer for the suggestions. Pulse distortion typically produces non-Markovian error since it depends on the previous gate played in the pulse sequence. RB fidelity is not a good merit for such error since it assumes time- and gate-independent error (Phys. Rev. Lett. 106, 180504 (2011), Phys. Rev. Lett. 109, 080505 (2012)).

We have added the measured transfer function and a comparison of a 6 ns flattop pulse before and after distortion, which we obtained directly from oscilloscope. This data gives a better visual comparison to elucidate that the predistortion is critical for pulses under 10 ns.

- Unexpected Leakage at 6.6ns

We noticed that at 6.6ns, leakage to the state $|3\rangle$ jumps by an order of magnitude. This is surprising because, according to Equation 2, the transition $1 \to 3$ should be much weaker than the $0 \to 2$ transition. What is driving this?

We thank the reviewer for the question. The strength of the transition $1 \to 3$ and $0 \to 2$ strongly depends on the pulse shape and the amplitude, which indicates the transition $1 \to 3$ is not always weaker than the $0 \to 2$. The numerical simulation results capture this feature well, and predict the increase of leakage to $|3\rangle$ at 6.6 ns in the experimental implementation as shown in Fig. 4.

Also, how are the solid lines (red/purple) being extracted? If these are simulations at optimal points satisfied by equations 5 and 6, shouldn't coherent errors theoretically be zero? If they aren't, which specific transitions are the main culprits?

We thank the reviewer for the valuable insights.

As discussed in the main text and the section "Single Qubit Gate Simulation" in the supplementary material, the solid lines are the optimal numerical simulation results from Nelder-Mead optimization on all three α parameters using total leakage error as the cost function in the absent of incoherent processes.

The increase of the leakage at the short pulse duration comes from multiple aspects. First, similar to other pulse shaping techniques, the leakage always exists for any pulse durations and rises up at shorter pulse durations, even for simulations without incoherent channels. This is mainly due to other unaddressed transitions. In our case particularly, we attribute this to the $|0\rangle \to |3\rangle$ transition as the pulse duration goes shorter. The correction for this transition is not considered during the calibration. Additionally, the diabatic error that is proportional to the derivatives becomes comparable to the relevant transition detuning in strong driving pulse. Moreover, the pulse that aligns well with the theoretical analysis may not be achievable in simulations and experiments. When pulse duration goes shorter, the optimal theoretical parameters that satisfies the Eq. 5 and 6 leads to negative values in Ω^2. As shown in Fig. S4, the parameters α_{02}, α_{13} can be adjusted to avoid this singularity by sacrificing the leakage suppression.

We also added the citation to arXiv:2512.19919 for the detailed analysis.

- APE Protocol Implementation

In the calibration protocol, the authors have used the amplifying phase error (APE) technique, and cited E. Lucero et al. "Reduced phase error through optimized control of a superconducting qubit", Physical Review A 82, 10.1103/PhysRevA.82.042339, (2010). After equation 3, the paper mentions that pi pulses cannot amplify error, as the phase error cancels. However, the authors have used this protocol, have they modified the protocol?

The APE protocol has been proven to work for X pulses experimentally. For example, in Phys. Rev. Lett. 116, 020501 (2016) Fig. 2, a reference cited in this manuscript, as well as in these works: Phys. Rev. X. 13, 031035 (2023) Fig. 10, and npj Quantum Inf. 9, 111 (2023) Fig. 4. We have added citations to these references.

To further aid readers, we added an additional section in the supplementary material to explain this.

In the conclusions, the authors mention that R2D helps with driving qubits simultaneously. Could they elaborate more on this? It's not clear whether they refer to general crosstalk or specific resonant transitions triggered by the drive on Qubit A affecting Qubit B?

We thank the reviewer for the comments. When applying gate operations in parallel to multiple qubits, the microwave crosstalk between the qubits will degrade the gate performances. Similar to leakage during the single qubit operation, such crosstalk effect is due to unwanted transitions during simultaneous gate operations. The existence and the suppression of multi-photon transitions demonstrated in this manuscript will give more insights to address the drive crosstalk.

We have reworded the statement to make this clearer.

Reviewer #4:

- What are the noteworthy results?

The paper by Y. Gao et al demonstrated a new pulse shaping technique for single qubit gates to suppress leakage and showcased very low leakage (below 2e-5) for 6.8 ns pi and pi/2 gate; the authors achieve such low leakage with a record value for the ratio of drive strength over qubit anharmonicity.

The paper also demonstrated Amplified Leakage Error (ALE) sequence that was used for both coherent leakage characterization and pulse parameter optimization; the technique was shown to be able to detect leakage as low as 1e-6.

- Will the work be of significance to the field and related fields? How does it compare to the established literature? If the work is not original, please provide relevant references.

Yes, the work is of significance to the field of superconducting qubits and superconducting quantum computing with a few caveats.

We are happy to see the reviewer considers our work significant. We thank the reviewer for providing us the valuable comments.

In terms of gate performance, for pi gates, the leakage number of 2e-5 for 6.8 ns gate is probably indeed the record for the same ratio of drive strength over qubit anharmonicity, as far as I know. However, experimental data in the paper shows significant increase in additional control error [Fig. 4c] as we go to short gates, which the authors attributed to pulse distortion. It is unclear how well we can mitigate this effect and it makes the practical usefulness of the pulse shaping technique questionable, at least for pi pulse.

We thank the reviewer for appreciating the record value we achieved for X gates. We do agree that there is indeed an increase in control error as we go to short gates. However, the increasing prominence of pulse distortion is a direct consequence of the successful suppression of other existing errors, such as multi-photon transitions. This gives valuable insights on the quantum control systems and arguably steers the research direction in this field.

First, this manuscript focuses on the suppression of the leakage error and understanding the physics of qubit under strong driving. The mitigation of pulse distortion is a topic on its own and arguably falls outside of the scope of this manuscript. Our work gives an explicit answer to the persistent problem about the root cause of the leakage in the strongly driven regime, and opens a clear path towards better gate performances by exposing the remaining

errors from decoherence and pulse distortion. This is exactly the driving force that motivates the field for further improvements. In fact, there are research groups that investigate pulse distortion at short pulse duration. For example, PRX Quantum 5, 030353 (2024), and F50-004 APS March Meeting 2024.

Second, there are calibration protocols that measure in time domain and require X operations, for example pulse distortion protocols in Phys. Rev. Lett. 110, 040502 (2013) and PRX Quantum 5, 030353 (2024). A shorter X gate gives higher time resolution which those protocols can benefit from.

Finally, the alternative of an X operation using double X/2 gates is worse than a single X gate. As shown in Fig. 4, double X/2 gates at 6.8 ns give an X operation that is 13.6 ns long with more than 2×10^{-4} total gate error, which is worse than a single X gate at 6.8ns in both speed and fidelity.

For pi/2 gate, the leakage number reported in the paper is on a par with previous work published in PRX Quantum 5, 030353 which achieved 2e-5 leakage for 6.25 ns gate and even smaller anharmonicity.

We do agree that both works are comparable in terms of gate duration and fidelity. However, in PRX Quantum 5, 030353 (2024), such result is achieved by introducing over 6 extra parameters more than the standard method and remains narrowly focused on single photon transitions. This is undesirable for punctual experimental calibration where the search space scales exponentially in the number of parameters. In fact, addressing the overlooked multi-photon transitions enables the use of only two semi-classical parameters to achieve record performances for both X and X/2 gates in our work. This is in stark contrast to heuristic modifications of the pulse spectrum, that provide neither insight into the physics nor a pathway to scalable implementation.

Regarding the ALE sequence used for leakage characterization in this paper, I would argue that this is not new. A similar technique was used in Phys. Rev. Lett. 100, 247001 for characterizing coherent leakage in pi pulse, dubbed as "ramsey error filter"; recently it was generalized to pi/2 pulse in Phys. Rev. Lett. 135, 130601. The pulse sequence is exactly the same as used in the paper by Y. Gao et al. The only difference is that Y. Gao et al used virtual Z gate to accumulate phase whereas the aforementioned two papers add wait time after the gate. In fact it was mentioned in Phys. Rev. Lett. 135, 130601 that virtual Z gate and adding physical wait time achieve exactly the same effect.

While indeed the protocols for leakage amplification are similar, it is not trivial to implement it with virtual Z instead of a time delay. We demonstrated that the leakage to $|2\rangle$ has three distinct amplification phases, which differs markedly from the prediction of two peaks in Phys. Rev. Lett. 135, 130601(2025). Moreover, we have generalized the protocol to higher leakage levels. Furthermore, this manuscript provides detailed theoretical analysis enabling the extraction of leakage error from this protocol, and the final leakage error could be extracted if and only if the protocol performs at all three phases, whereas Phys. Rev. Lett. 135, 130601(2025) fails to extract the actually value of leakage error, and consequently limited to a qualitative calibration.

With these said, I do believe the results of the paper, in particular, the pulse shaping technique is still of significance to the field. However, given the caveats I listed above, I do not think the paper suits a prestigious article as Nature Communications.

We strongly believe that the identification of the critical role of multi-photon transitions in our work both elucidates the physics of strong driving, a foundational topic in qubit control that has thus far been ignored, and uses this to establish a novel platform for quantum control, enabling a record gate fidelity. Our manuscript is the first control solution to the removal of multi-photon, virtual transitions ever demonstrated, not just in superconducting qubits, but even in optics where such processes are ubiquitous. We believe that the results presented in this manuscript will have a critical and profound impact on the field.

Does the work support the conclusions and claims, or is additional evidence needed?

Yes, the work supports the conclusions and claims.

- Are there any flaws in the data analysis, interpretation and conclusions?

- Do these prohibit publication or require revision?

There are a few places in the paper that require clarification and minor revision.

1) Expressions for lambda's need to be given

We have done as the reviewer asks.

2) It would be useful to comment why we need the fourth derivative in Eq. (3), that is, would we achieve similar performance by setting α_{02} or α_{13} to zero.

We thank the reviewer for valuable comments. The Fig. 4(e-f) in this manuscript indicates that $|1\rangle \rightarrow |3\rangle$ transition has less impact at longer gate duration and can be omitted. However, as shown in Fig. 1 and 8 of arXiv.2512.19919, when we only use α_{02} by setting α_{13} to 0, the leakage to $|3\rangle$ becomes the limiting factor in the ultrafast pulses. We do believe that in order to achieve the shortest gate with low leakage, both corrections need to be included in the pulse shaping. Please see arXiv.2512.19919 for more details. We have added additional comments in the main text to address this.

3) The matrix for X in Eq. (7) does not appear to be unitary. Can the authors comment on why?

We thank the reviewer for spotting the typo. The element of third row second column should be $-A_1 e^{i(\phi_1 - \phi_2)}$ in Eq. 7. After the correction, the matrix is unitary up to the first order, where $XX^\dagger = I + O(A^2)$

- Is the methodology sound? Does the work meet the expected standards in your field?

Yes and yes

- Is there enough detail provided in the methods for the work to be reproduced?

Yes

REPLY TO REVIEWERS

Reviewer #1:

I appreciate that the authors have revised the manuscript in accordance with my suggestions. They have also addressed all the comments from the reviewers, and the overall quality of the paper has improved. The only remaining issue concerns the relationship between Figs. 2(c) and (e) and Fig. 3(f), which is still unclear. Specifically, Fig. 3(f) shows five curves, whereas Fig. 2(c) contains four peaks. This discrepancy needs further clarification and explanation. In my view, after addressing the point mentioned above, the manuscript meets the criteria for publication in Nature Communications.

We appreciate the positive assessment and recommendation from the reviewer. In particular, we thank the reviewer for pointing out this issue. In Fig. 2(b), the amplification phases are at $\theta_k^{|2\rangle} = (0.35 + 2k/3)\pi,\ k = 0\ldots2$, which are clearly visible. In Fig. 2(c), the amplification phases are at $\theta_k^{|3\rangle} = (0.22 + 2k/5)\pi,\ k = 0\ldots4$, of which the peak at $\theta_4^{|3\rangle}$ is only slightly above the noise floor, as it has not reach its oscillation period within 300 repetitions. This agrees with the theoretical prediction that the frequency and the amplitude of the population oscillation at those amplification phases are generally distinct.In Fig. 3(e)-(f), we plot the population oscillation curves at all amplification phases $\theta_k^{|2\rangle}$ and $\theta_k^{|3\rangle}$ (three phases for $|2\rangle$, and five phases for $|3\rangle$). Here, the maximum population at $\theta_0^{|2\rangle}$ and $\theta_2^{|3\rangle}$ are lower. We have added more descriptions in the main text and also modified the Fig. 2(b)-(c) and Fig. 3(e)-(f) to better clarify this.

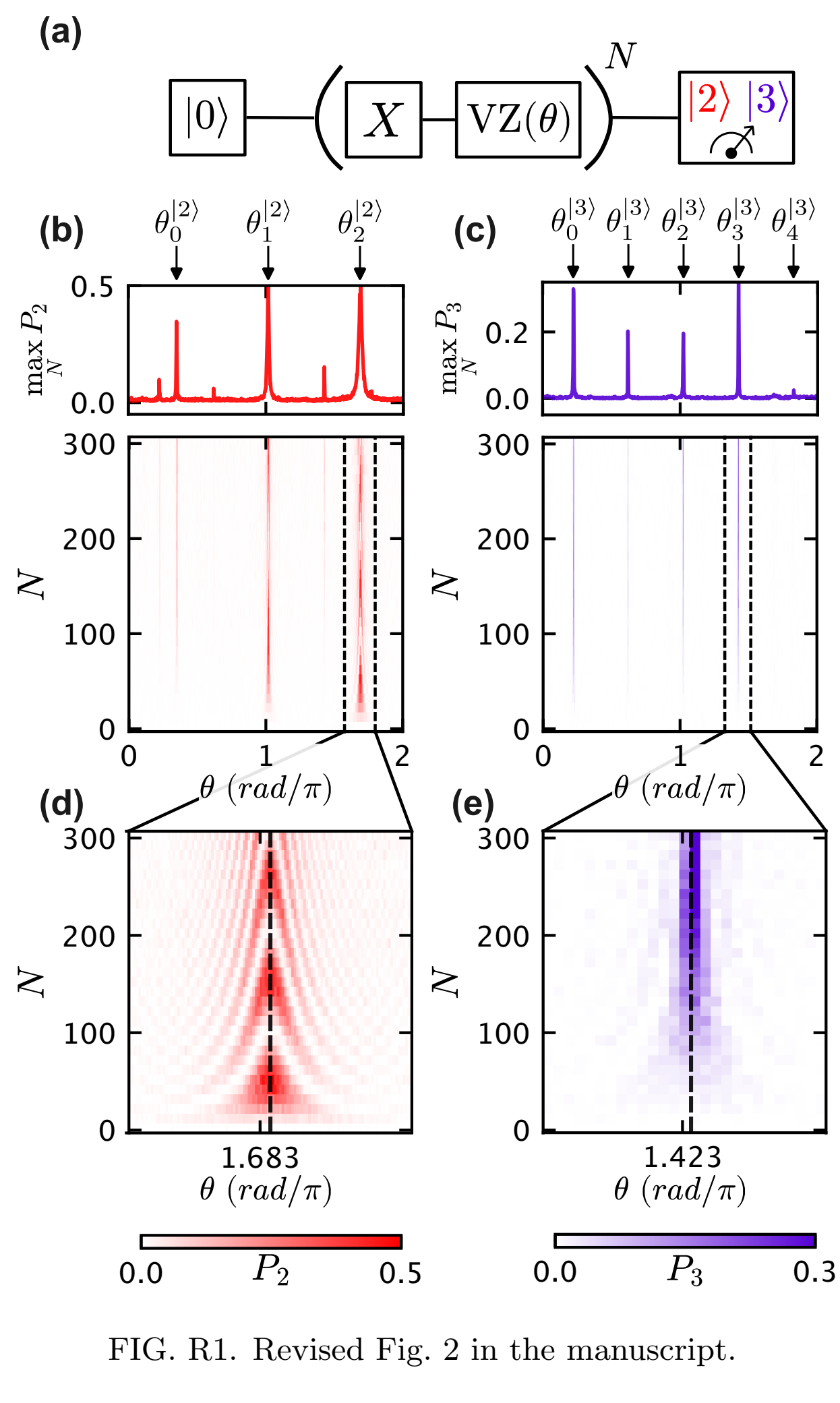

FIG. R1. Revised Fig. 2 in the manuscript.

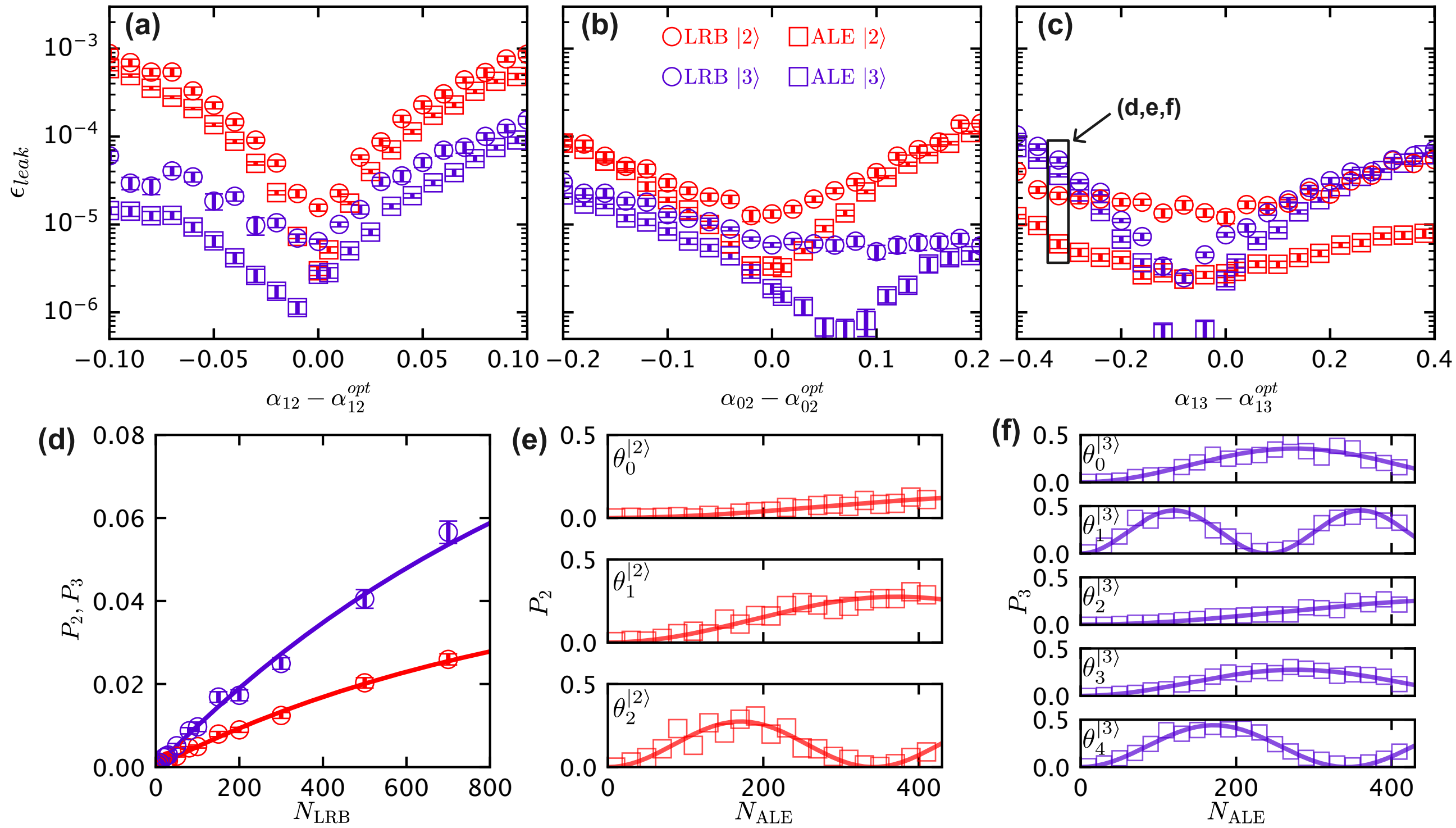

FIG. R2. Revised Fig. 3 in the manuscript.

Reviewer #2:

After careful consideration, we remain unconvinced that this manuscript meets the level of novelty and impact expected for publication in a high-profile journal such as Nature Communications. We would therefore recommend transfer to NPJ Quantum Information or consideration for publication in other specialized journals, such as PRX Quantum. While we appreciate the authors' approach and acknowledge the arguments presented, we do not consider the underlying concepts to be sufficiently novel. In particular, the idea of employing pulse shapes involving multiple derivatives to suppress undesired transitions has previously been introduced by Prof. F. Motzoi and collaborators in the context of the cross-resonance gate (Box Li at al, npj Quantum Information volume 10, Article number: 66 (2024)). The recursive extension of the DRAG formalism proposed in the submitted manuscript, aimed at simultaneously suppressing multiple leakage and error transitions, appears conceptually very similar to the methodology developed in that earlier work.

Although the manuscript provides a useful generalization and demonstrates the applicability of the approach in a different setting, we do not believe that the conceptual advance is substantial enough to justify publication in Nature Communications.

We thank the reviewer for taking time to review this work and providing us valuable comments and assessments. We agree that derivative-based suppression of transitions has important precedents. However, the advance of the present work is not the abstract use of multiple derivatives. Rather, we experimentally identify the individual multiphoton processes that fundamentally limit ultrafast single qubit gates, derive a channel-specific second-derivative correction from the corresponding strong-drive effective Hamiltonian, and develop a level-resolved calibration protocol that permits the corrections to be independently and systematically optimized.

From a theoretical perspective, there are also fundamental differences between our approach and Li et al. Their work addresses the highly off-resonant transitions that completely commute with the driven dynamics while neglecting many non-commutative effects that arise in the full multilevel dynamic. In contrast, our accompanying theoretical manuscript (arXiv:2512.19919) presents a detailed derivation demonstrating how these non-commuting terms can be systematically incorporated into the effective Hamiltonian. While there are perceptual similarities in the pulse shapes, the pulse design proposed by Li et al. cannot be directly applied to the strongly driven multilevel system considered here. To definitively demonstrate this, we have applied the pulses from Li et al. to our setup (Eq. R1 and Eq. R2)

and show numerically that this approach fails to achieve a high fidelity X gate, as shown in Fig. R3.

$$\Omega_1^2 = (1 - i\frac{\alpha_{02}}{\Delta}\frac{d}{dt})(1 - i\frac{\alpha_{13}}{3\Delta}\frac{d}{dt})\Omega_0^2 \tag{R1}$$

$$\Omega = (1 - i\frac{\alpha_{12}}{\Delta}\frac{d}{dt})\Omega_1 \tag{R2}$$

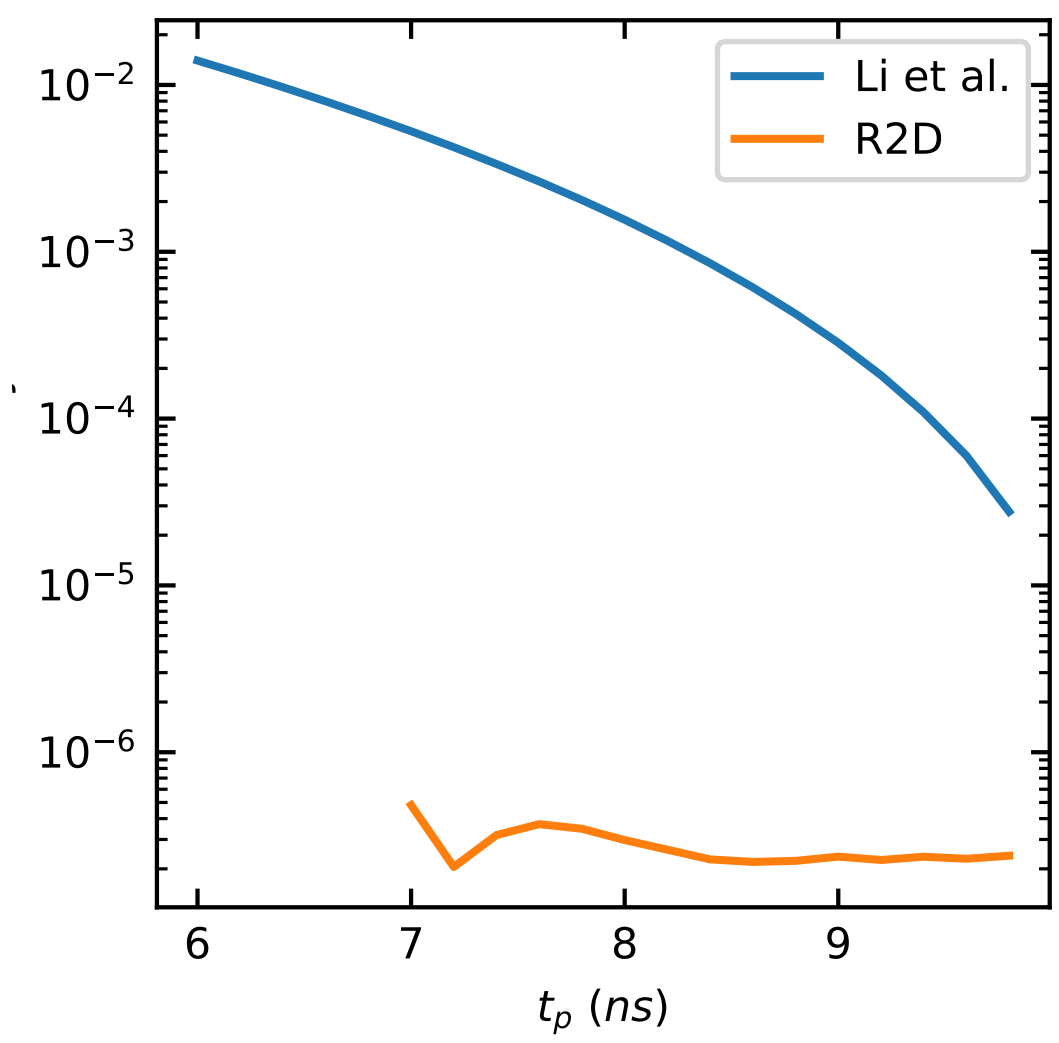

FIG. R3. Numerical simulation of gate errors in absence of decoherence.

Experimentally, we identify multiphoton leakage as a dominant error mechanism in high-fidelity, sub-7-ns resonant single-qubit gates, and simultaneously suppress the leakage from $|0\rangle \rightarrow |2\rangle$ and $|1\rangle \rightarrow |3\rangle$. The combination of physical identification, targeted control, and quantitative calibration is essential: it allows us to demonstrate that the observed multiphoton channels are causal limitations on gate performance and that their removal brings the gates into a predominantly decoherence-limited regime. Moreover, this manuscript experimentally illustrates the significant influence of higher energy levels, like $|3\rangle$, in ultrafast single qubit gates. While higher energy levels are routinely included in numerical Hamiltonian simulations, to the best of our knowledge no previous experimental work has independently calibrated leakage into these higher levels or demonstrated that addressing such leakage leads to measurable improvements in single-qubit gate fidelity.

Overall, our work has not only validated the predictions of the higher-order theoretical framework (arXiv:2512.19919), but enables a first-in-kind demonstration of strong driving in weakly anharmonic qubits.

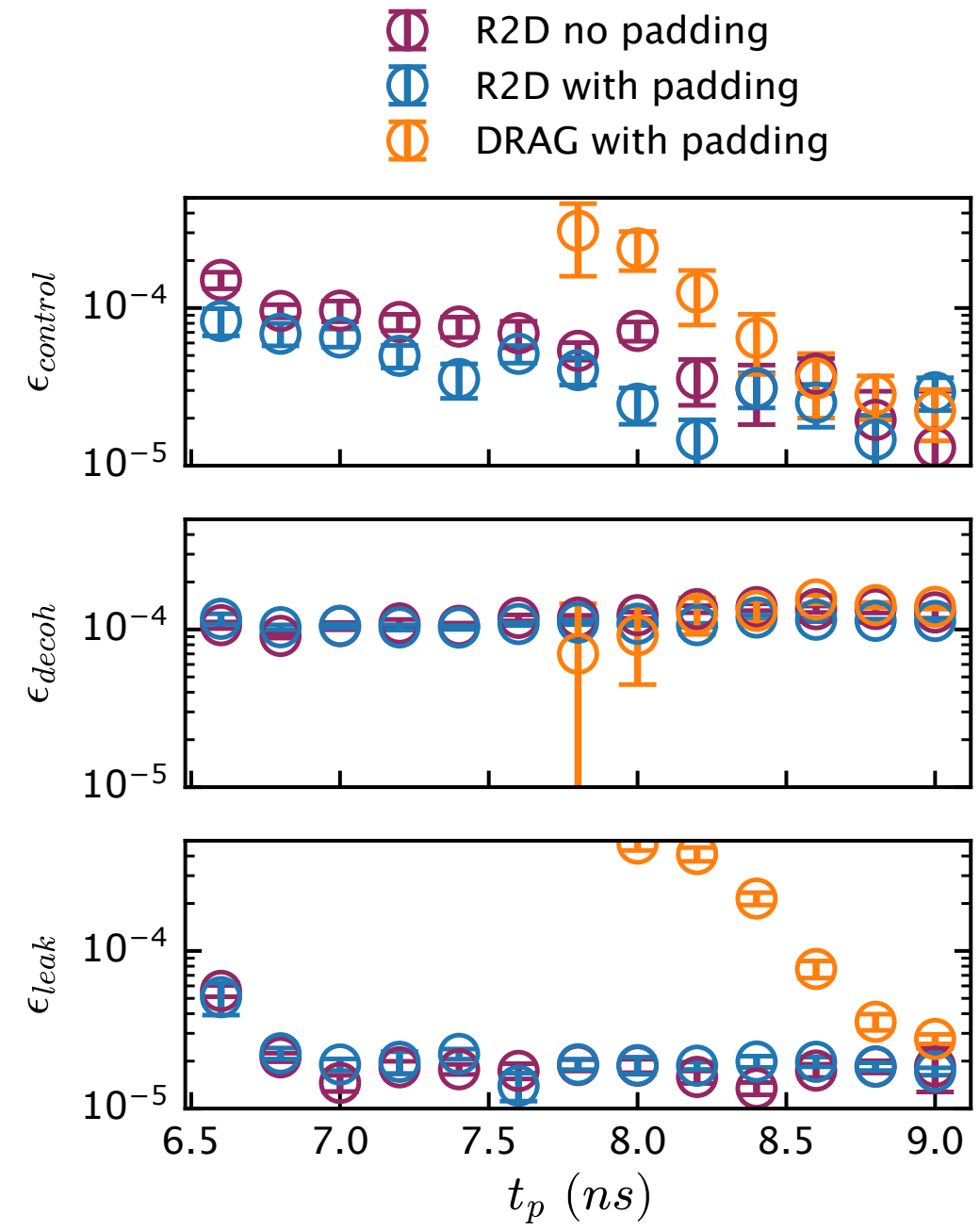

FIG. R4. The error budgets for the R2D and DRAG pulse shapes. The padding reduces control error (top) by $1 \sim 4 \times 10^{-5}$, and does not influence the decoherence error (middle) and the leakage error (bottom).

Reviewer #4:

I do not find the responses from the authors completely satisfactory.

1) Regarding the practical usefulness of the technique, as shown in Fig. 4c), due to the increase in the control error, the general trend is that total gate error increases as gate duration becomes smaller. Because of this, it is unclear how practically useful it is to have single qubit gates with duration below 10 ns.

We do agree that total gate error increases as the gate duration becomes smaller in our manuscript. However, the findings presented in this manuscript still have their significance and practical usefulness.

First, for real algorithms, single-qubit gates are typically applied only to the qubits involved in the intended operation, while other qubits may need to remain idle, leading to decoherence error for the entire circuit. Although compilers maximize parallel gate execution to reduce circuit depth, idle periods are unavoidable. The larger the processor is, the more idle periods exist. Reducing the duration of single-qubit gates is beneficial because it shortens the overall circuit execution time and decreases the exposure of idle qubits to decoherence mechanisms. As shown in Fig. 4(c) of the manuscript, the total gate error only increases less than 5×10^{-5} while reducing pulse duration from 9 ns to 7 ns. In large scale processors where the T_1 and T_2 are generally around 70 μs (Nature volume 638, pages 920–926 (2025) the Willow, Phys. Rev. Lett. 134, 090601 (2025) the Zuchongzhi 3.0, arXiv:2408.12433 (2024) the IQM Garnet), this corresponds to a reduction of 1.4×10^{-5} error **per idling qubits** ($\epsilon = t/6T_1 + t/3T_2$, Phys. Rev. Applied 3, 044009 (2015)). Thus, our faster single qubit gates improve the balance between decoherence error and control-induced error across the whole chip, especially for large scale processors.

Furthermore, although the manuscript shows a moderate increasing trend in control infidelity, such degradation is not necessarily fundamental and can be further mitigated through future advances in control electronics, improved microwave delivery systems, and more accurate characterization and compensation of pulse distortions. In contrast, leakage errors are fundamentally constrained by the pulse waveform and the multilevel nature of superconducting qubits, particularly when operating in the regime of strong driving. Therefore, addressing the intrinsic limitations imposed by pulse shapes and suppressing leakage at the source is essential for scalable quantum processors. In this manuscript, we focus on optimizing the pulse design to overcome these fundamental leakage limitations while maintaining high gate performance.

We have included this practicality in the conclusion of the manuscript, we would like to thank the reviewer again for bringing up this point.

The authors emphasize that the goal of the technique is to suppress leakage error, mitigation of pulse distortion is beyond the scope of the work. I do not find this answer fully satisfactory because it is not very clear where exactly the additional control error is coming from. There is a possibility that the additional control error is a consequence of applying the R2D pulse.

We thank the reviewer for raising this valid concern about the source of the control error. In Fig. 2 and 8 of arXiv:2512.19919, which is cited in the manuscript, the coherent simulation shows R2D pulse shape has the ability to achieve total error rates below 10^{-5} in absent of decoherence error. If the source of the control error is the pulse shaping, it will be captured by this coherent simulation.

To further address this concern, we include an additional figure, as in Fig. R4 of this response, where we compare the error budgets between different pulse configurations. Comparing the curves R2D with padding (blue symbols) and DRAG with padding (orange symbols), we see that introducing R2D pulse shaping does not increase the control error, but rather decreases it. In addition, the correlated increasing trend of control and leakage error observed when using DRAG, which is absent when using R2D, suggests that the reduction in leakage achieved by introducing R2D improves the calibration accuracy.

Furthermore, Fig. R4 shows that the padding after the pulse duration effectively decreases the control error while showing no influence on decoherence error and leakage error (blue and purple symbols). This feature matches well with the notion that control error is caused by pulse distortions. If the additional control error is a consequence of the R2D pulse shape, such error would be confined within the pulse duration and should not be affected by the padding after the pulse. We have included these figures in the supplementary material in the revised version.

Pulse distortions commonly exist in any electrical systems, caused by control electronics and wiring in the fridge, and it becomes detrimental especially for shorter pulses. The research community is at present developing the next generation of electronics, wiring solutions, as well as better methodology for microwave distortion characterization (Rev. Sci. Instrum. 96, 104709 (2025), Nature Electronics volume 9, pages 287–294 (2026), PRX Quantum 5, 030353 (2024)), we therefore believe this control error caused by imperfection of the signal delivery would be eased in the future, further increasing the impact and practicality of this ultrafast pulses.

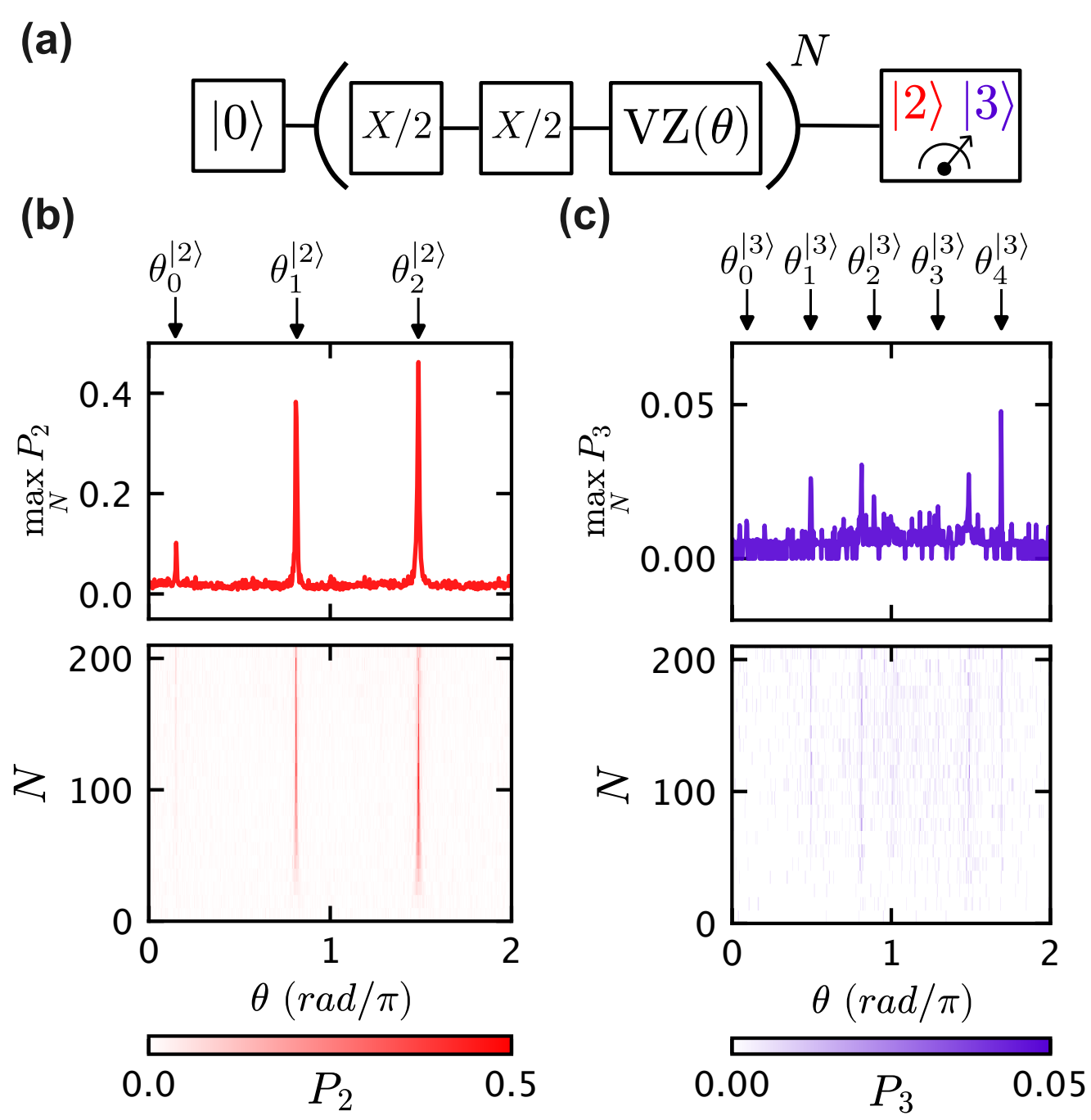

FIG. R5. ALE results for $X/2$ gates. (a) The pulse sequence. (b) The $|2\rangle$ leakage population as a function of θ and N. The line plot on top shows the maximum leakage population for each θ. The amplification phases are at $\theta_k^{|2\rangle} = (0.153{+}2k/3)\pi,\ k = 0\dots2$. (c) Same as (b) but plotted with $|3\rangle$ leakage population. The amplification phases are at $\theta_k^{|3\rangle} = (0.09 + 2k/5)\pi,\ k = 0\dots4$.

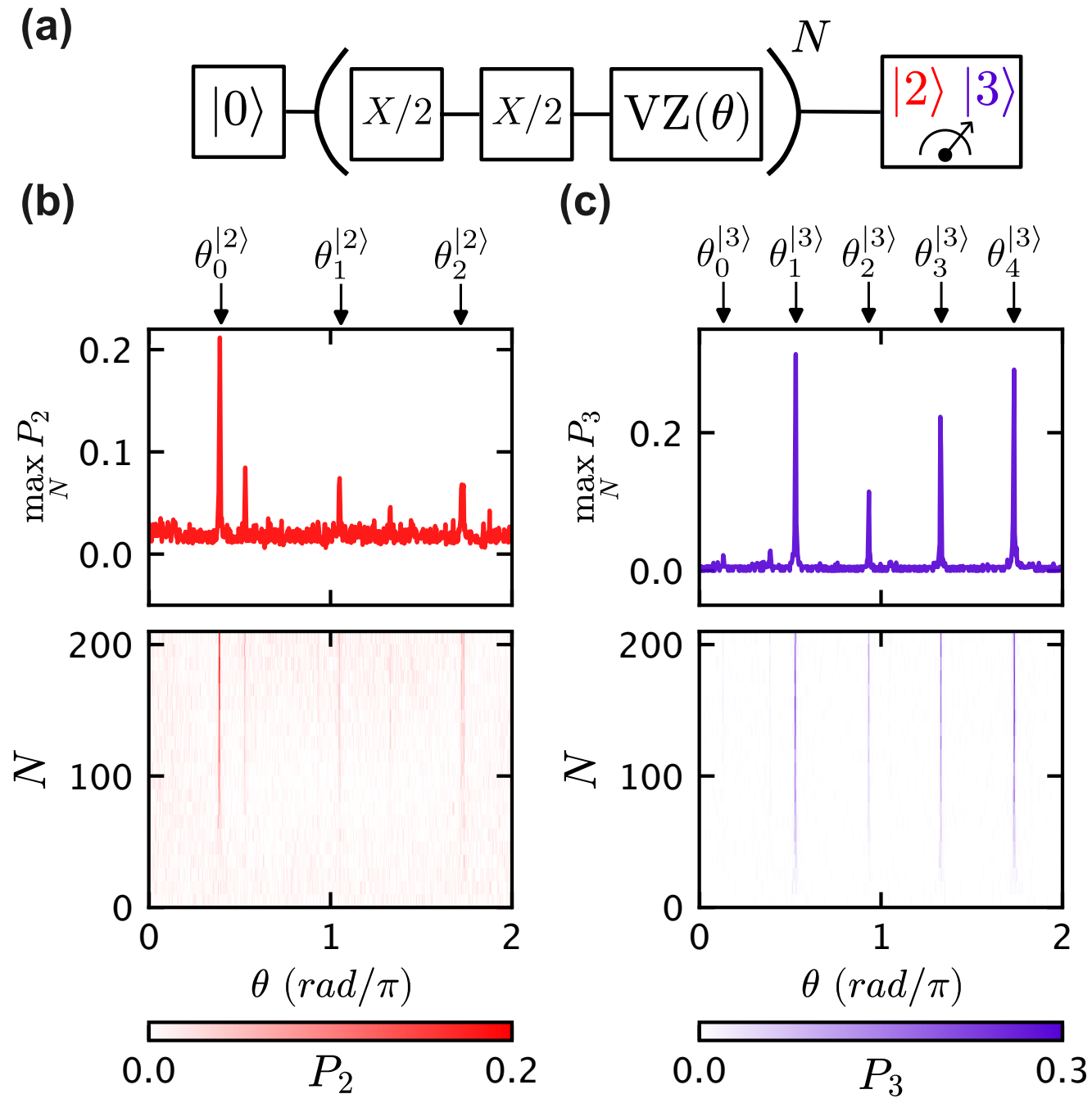

FIG. R6. ALE results for $X/2$ gates. (a) The pulse sequence. (b) The $|2\rangle$ leakage population as a function of θ and N. The line plot on top shows the maximum leakage population for each θ. The amplification phases are at $\theta_k^{|2\rangle} = (0.387{+}2k/3)\pi,\ k = 0\ldots2$. (c) Same as (b) but plotted with $|3\rangle$ leakage population. The amplification phases are at $\theta_k^{|3\rangle} = (0.13 + 2k/5)\pi,\ k = 0\ldots4$.

2) Regarding the similarity between the ALE technique used by the authors and the technique used in PRL 100, 247001 and PRL 135, 130601 (2025), I also do not find the authors' response completely satisfactory. The authors stated that they observed three distinct amplification phases while PRL 135, 130601 only predicted two. The authors were probably referring to their results for pi pulse in Fig. 2b which show three distinct amplification phases. However, results in PRL 135, 130601 (2025) were for pi/2 pulses. They do not necessarily have the same number of amplification peaks as for pi pulse. It would be more convincing if authors also show ALE results for pi/2 pulses which I do not find in the current manuscript.

We understand the concern from the reviewer, we therefore include two distinct datasets of ALE results for $X/2$ gates, as shown in Fig. R5 and Fig. R6 in this response.

In Fig. R5(b), the three amplification phases are clearly visible, and they are evenly spaced in 2π period. In Fig. R5(c), only two of five peaks $\theta_1^{|3\rangle}$ and $\theta_4^{|3\rangle}$ are clearly visible due to low leakage rate to $|3\rangle$. Fig. R6 shows a different dataset for $X/2$ with different pulse parameters and duration, where the leakage to $|3\rangle$ is higher. In Fig. R6(c), four of five peaks are clearly visible, the remaining one at $\theta_0^{|3\rangle}$ is only slightly above noise level, and the peaks are evenly spaced by $2\pi/5$.

The number of amplification phases and their spacing in both figures match well with the theory prediction in the supplementary material. We have included these figures in the supplementary material in the revised version.

In this paper, the authors experimentally demonstrated ultrafast and high-fidelity single qubit gates by using R2D method. The authors also introduced a method that can precisely quantify leakage rates. Improving gate operation is very important, and the present work addresses this relevant and timely topic. The measurements appear to have been carried out carefully, and the quality of the data is good enough.

In my view, the paper has the potential to meet the publication criteria of Nature communications. However, there remain several points that should be clarified and explained more explicitly to improve the clarity and accessibility of the paper for the reader, as detailed below.

issues

1. Fig. 1(b): "vertical solid line" would be better for the unwanted transition frequencies.

2. Figs. 2 and 3:

   What are the three lines in Fig. 3(e)? Is one of the curves in Fig. 3(e) cross section of the plot in Fig. 2(d)? What are the three lines in Fig. 3(f)? Is one of the curves in Fig. 3(f) cross section of the plot in Fig. 2(e)? The relation between them should be clarified.

3. lines 241-244: The authors attribute the difference in leakage errors to the fact that the ALE method is insensitive to thermal excitations and $|2\rangle \rightarrow |3\rangle$ transition errors. The authors have to describe the reasons by referring Figures or equations.

I appreciate that the authors have revised the manuscript in accordance with my suggestions. They have also addressed all the comments from the reviewers, and the overall quality of the paper has improved. The only remaining issue concerns the relationship between Figs. 2(c) and (e) and Fig. 3(f), which is still unclear. Specifically, Fig. 3(f) shows five curves, whereas Fig. 2(c) contains four peaks. This discrepancy needs further clarification and explanation. In my view, after addressing the point mentioned above, the manuscript meets the criteria for publication in Nature Communications.
